# Longitudinal characterization of *Gaa*^c.1826dupA^ mice reveals the cardiac, myopathic and biochemical phenotypes of Pompe disease

**DOI:** 10.1242/dmm.052611

**Published:** 2026-03-18

**Authors:** Jerry F. Harb, Shih-hsin Kan, Chloe L. Christensen, Allisandra K. Rha, Perla Andrade-Heckman, Agatha Kliman, Alejandra Padilla, Cora Holbrook, Jeffrey Y. Huang, Dwight D. Koeberl, Raymond Y. Wang

**Affiliations:** ^1^ Research Institute, Children's Hospital of Orange County, Orange, CA 92868, USA; ^2^ Department of Pediatrics, University of California-Irvine School of Medicine, Irvine, CA 92697, USA; ^3^ Pediatric Cardiac Lab, Loma Linda University Children's Health, Loma Linda, CA 92354, USA; ^4^ Department of Pediatrics, Duke University School of Medicine, Durham, NC 27710, USA; ^5^ Department of Molecular Genetics and Microbiology, Duke University Medical Center, Durham, NC 27710, USA; ^6^ Division of Metabolic Disorders, Children's Hospital of Orange County Specialists, Orange, CA 92868, USA

**Keywords:** Lysosomal storage disorder, Acid α-glucosidase, GAA deficiency, Glycogen storage, Murine model, Natural history

## Abstract

Pompe disease (PD) is a rare autosomal recessive disorder caused by acid α-glucosidase (GAA) deficiency, leading to lysosomal glycogen accumulation. Pathogenic GAA variants result in enzyme dysfunction and glycogen storage in cardiac, skeletal and smooth muscle, as well as in the central nervous system, driving both systemic and neurological manifestations. We have previously characterized a transgenic knock-in (KI) mouse carrying the *Gaa* c.1826dupA variant to 12 weeks of age, showing that it recapitulates key biochemical and phenotypic features of PD. Here, we extend this analysis to present a long-term characterization of this *Gaa* c.1826dupA KI mouse model by using physiological, behavioral, biochemical and histopathological assessments. KI mice exhibited early-onset hypertrophic cardiomyopathy with significant cardiac functional decline, reduced body mass, impaired skeletal muscle strength, locomotion, coordination and balance. Biochemically, KI mice showed decreased GAA activity and increased lysosomal glycogen accumulation in the heart, diaphragm, gastrocnemius and brain. Despite these abnormalities, survival did not differ from wild-type mice – a divergence from severe human PD but consistent with other murine models. Collectively, these findings support this KI model as a translational platform for therapeutic evaluation in PD.

## Teaser

**Summary:** This comprehensive natural history of a Pompe disease mouse model comprising a human-relevant mutation shows that it recapitulates key disease features, thereby, supporting its use in preclinical therapeutic testing.

## INTRODUCTION

Pompe disease (PD) is a rare autosomal recessive disorder that results in lysosomal glycogen accumulation due to a deficiency in the enzyme acid α-glucosidase (GAA; also known as acid maltase). Between 2010 and 2022, over 11.6 million newborns were screened for PD across 22 states and eight countries on four continents (US, Europe, Latin America, Asia), and the reported global birth prevalence of PD was 1:18,711 (∼5.3 cases per 100,000 live births) (Colburn and Lapidus, 2023). In affected individuals, biallelic alterations in *GAA* result in deficient GAA enzyme and lead to the build-up of glycogen within tissues, with the most prominent effects on cardiac, skeletal and smooth muscle (Kishnani and Howell, 2004). In addition to peripheral tissues, glycogen accumulation has also been described in the central nervous system (CNS), where it contributes to neurological manifestations of the disease (Korlimarla et al., 2019).

Infantile-onset Pompe disease (IOPD) presents with a more-severe disease course than late-onset Pompe disease (LOPD) (Slonim et al., 2000). IOPD presents within the first few months of life, with the most salient clinical manifestations being muscle weakness, hypertrophic cardiomyopathy (HCM), respiratory failure and hypotonia, among other symptoms. Without treatment, affected individuals succumb to this condition within the first year of life (Kishnani et al., 2006a; van den Hout et al., 2003). The attenuated form, LOPD, typically progresses at a slower rate and can manifest at any point during childhood (after 12 months), adolescence or adulthood. While individuals with LOPD do not exhibit the severe cardiac involvement observed in IOPD, they are prone to myopathy and, without treatment, ultimately succumb to respiratory complications (Stevens et al., 2022).

Intravenous recombinant human acid α-glucosidase (rhGAA) enzyme replacement therapy in conjunction with a high-protein diet is currently the standard of care for PD (Amalfitano et al., 2001; Kishnani et al., 2006b; Klinge et al., 2005; van den Hout et al., 2004). By utilizing the capability of cells to internalize lysosomal enzymes via the mannose-6-phosphate receptor, rhGAA is endocytosed and transported to lysosomes, enabling the degradation of accumulated glycogen (Roig-Zamboni et al., 2017).

Currently a murine model of PD, the B6;129-*Gaa^tm1Rabn^*/J mouse, is available (The Jackson Laboratory; RRID:IMSR_JAX:004154). However, the genetic modification for this transgenic knockout (KO) model – the full deletion of exon 6 – lacks human orthologues (Raben et al., 1998, 2010). We have previously developed a novel transgenic murine model of IOPD that harbors the *Gaa* c.1826dupA pathogenic variant (Huang et al., 2020). Unlike previous models, our knock-in (KI) model preserves the endogenous *Gaa* gene except for the c.1826dupA variant of interest. This clinically relevant, patient-derived mutation (rs754952153) is orthologous to human IOPD and introduces a premature stop codon (p.Y609ter) that abolishes functional GAA production, thereby recapitulating the profound enzymatic deficiency observed in patients with this variant. Although relatively rare, c.1826dupA has been reported in multiple IOPD cases and exemplifies the type of severe loss-of-function *GAA* mutations that drive early disease onset (Fukuhara et al., 2018; Mori et al., 2017; van den Hout et al., 2003). While both KO and KI models provide crucial insights into disease pathology, KI models, such as the IOPD model we present here, offer greater translational relevance by enabling the study of precise genetic alterations, ultimately giving a more accurate representation of the human condition.

In this study, we provide a comprehensive longitudinal evaluation of *Gaa*^c.1826dupA/c.1826dupA^ mice. Mice were assessed over an 84-week period, with physiological, behavioral, biochemical and histopathological analyses used to evaluate the differences between *Gaa*^+/+^ (wild type, WT) and the KI model. This study characterizes the natural history of the KI murine model and emphasizes its similarities to the human IOPD counterpart.

## RESULTS

### Study design – natural history characterization of the murine PD KI model

**Fig. 1.**
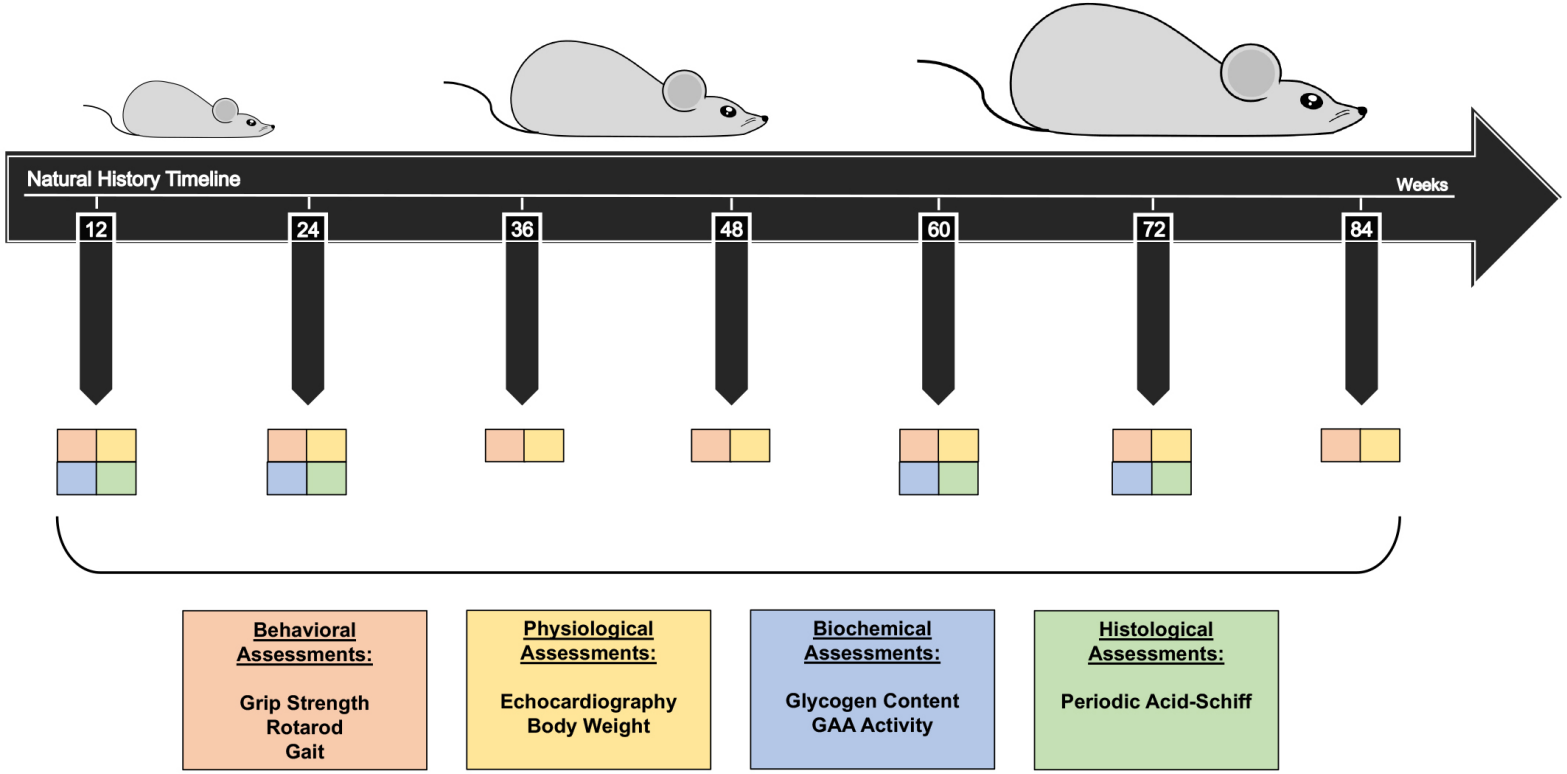
**KI murine model long-term natural history characterization.** The study design includes seven timepoints as indicated; assessments were conducted every 12 weeks, starting at 12 weeks of age and concluding at ∼84 weeks of age. Behavioral (orange) and physiological (yellow) evaluations were performed at each timepoint, while biochemical (blue) and histological (green) analyses were carried out at 12, 24, 60 and 72 weeks.

This study aims to characterize the long-term natural history of PD in a KI mouse model compared to WT controls. Mice were assessed longitudinally from 12 to 84 weeks of age at 12-week intervals (Fig. 1). Each cohort underwent *in vivo* assessments conducted at each timepoint to capture functional and physiological aspects associated with lysosomal glycogen accumulation. Physiological measures, including echocardiography and body weight tracking, were performed to assess cardiac function and overall health status. Behavioral assessments included gait analysis, forelimb muscle grip strength and rotarod performance to monitor neuromuscular impairment over time. Physiological measures, including echocardiography and body weight tracking, were performed to assess cardiac function and overall health status. To evaluate biochemical and histopathological changes, animals were harvested at 12, 24, 60 and 72 weeks of age for post-mortem analysis. Tissue collection enabled the quantification of glycogen content and GAA enzyme activity, while histopathological characterization was performed using periodic acid–Schiff (PAS) staining to visualize glycogen deposition. These assessments provided comprehensive insight into the progressive impact of PD in the KI model, offering a detailed characterization of disease onset, trajectory, key phenotypes and severity relative to WT controls. Both male and female mice were included in all experimental cohorts, with analyses incorporating sex as a biological variable. Aside from a difference observed in rotarod performance, no significant sex-specific effects were detected (Figs S1, S2, S3; Tables S1, S2). Results are, therefore, presented with sexes combined unless otherwise noted.

### Long-term echocardiographic assessment of the KI murine model demonstrates abnormal cardiac structure and function compared to WT mice

**Fig. 2.**
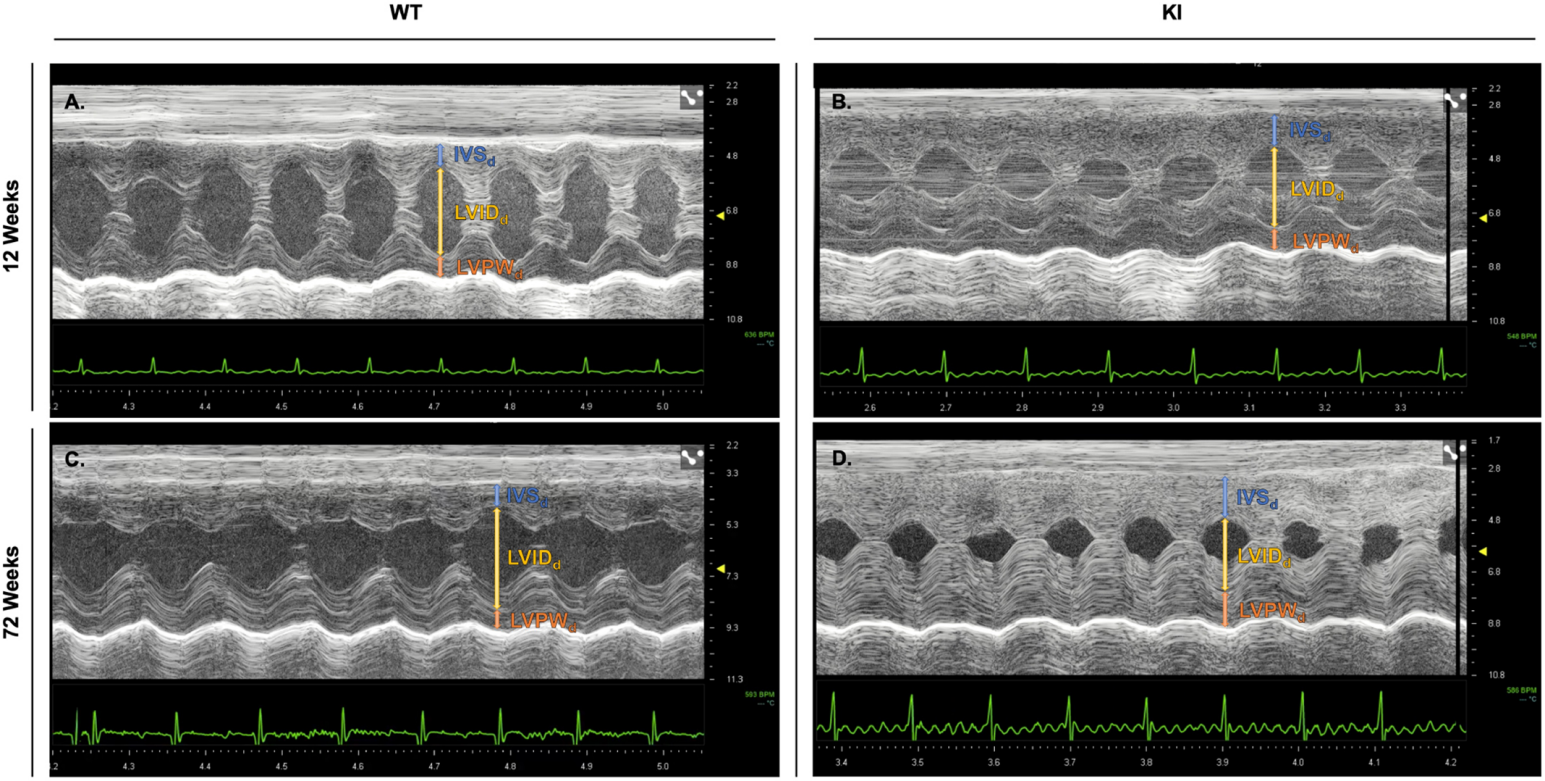
**Echocardiographic traces reveal differences in left ventricular structure between WT and KI mice at different timepoints.** (A-D) Representative M-mode echocardiographic imaging in the parasternal short axis view of WT (A) and KI (B) mice at 12 weeks, and of WT (C) and KI (D) mice at 72 weeks show thickened cardiac walls and reduced left ventricular internal diameter at end-diastole in KI compared to WT mice. Vertical lines indicate measurements of interventricular septum (IVS_d_; blue), left ventricular internal diameter (LVID_d_; yellow) and left ventricular posterior wall (LVPW_d_; orange) at end diastole.

Echocardiography, a non-invasive ultrasound imaging technique, was used to evaluate various aspects of cardiac health. Compared to WT mice, differences in cardiac structure and function were evident in KI mice as early as 12 weeks (Fig. 2A,B), while long-term evaluation demonstrates increasing KI cardiac hypertrophy (Fig. 2C,D). The following parameters were utilized as markers of heart health: interventricular septum (IVS) thickness, left ventricular posterior wall (LVPW) thickness, left ventricular mass/body weight [LVM/BW; hereafter referred to as left ventricular mass index (LVMI)], left ventricular internal diameter (LVID) and percentage of fractional shortening (%FS) at end diastole. LVID was also evaluated at end systole (LVID_s_).

**Fig. 3.**
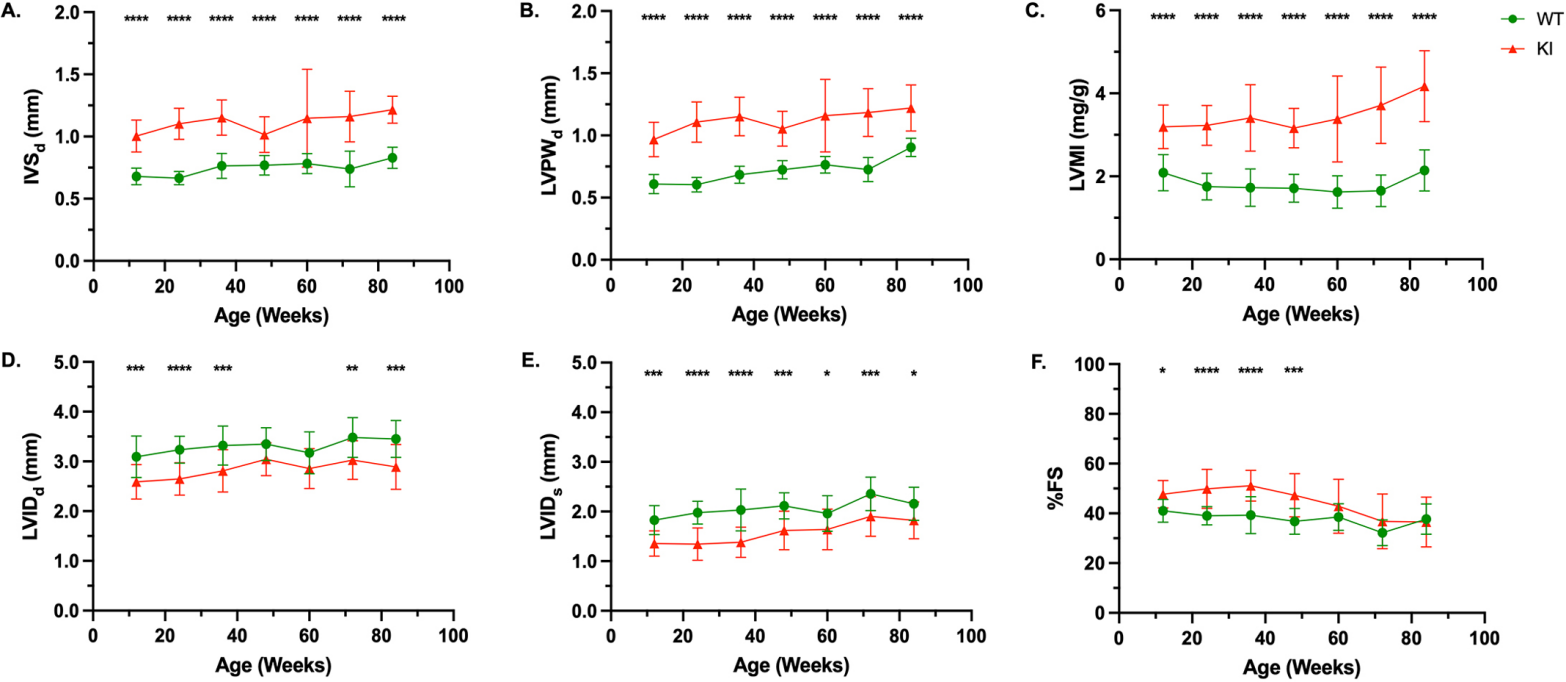
**Comprehensive cardiac evaluation of WT and KI mice.** (A-F) Comparative analysis of end-diastolic interventricular septum (IVS_d_) (A), end-diastolic left ventricular posterior wall (LVPW_d_) (B), left ventricular mass index (LVMI) (C), end-diastolic left ventricular internal diameter (LVID_d_) (D), end-systolic left ventricular internal diameter (LVID_s_) (E) and percentage of fractional shortening (%FS) (F) reveals progressive deterioration of cardiac parameters in KI mice over 84 weeks. Sample sizes (WT/KI): 12 weeks: *n*=20/21; 24 weeks: *n*=18/18; 36 weeks: *n*=18/18; 48 weeks: *n*=18/18; 60 weeks: *n*=17/18; 72 weeks: *n*=17/18; 84 weeks: *n*=16/18. Data were obtained from at least three independent experiments and are shown as the mean±s.d. All comparisons were analyzed using two-way ANOVA with Šídák post-hoc test. *P<0.05, **P<0.01, ***P<0.001, ****P<0.0001.

Structural parameters (IVS, LVPW, LVMI and LVID) demonstrated significant differences between WT and KI mice up to 84 weeks of age. KI mice exhibited increased IVS_d_ (Fig. 3A) and LVPW_d_ (Fig. 3B) as well as an elevated LVMI (Fig. 3C) compared to WT mice. The increase in IVS and LVPW wall thicknesses subsequently impacted LVID, with KI mice exhibiting a consistent decrease in LVID during both diastolic and systolic phases over the 84-week assessment. This reduction reached statistical significance at all timepoints during systolic phase and most timepoints during diastolic phase (Fig. 3D,E). %FS is a key parameter for assessing left ventricular systolic function, calculated as the percent reduction in LVID from diastole to systole, reflecting myocardial contractility. An elevated %FS indicates HCM, as thickened ventricular walls necessitate increased contractility to maintain cardiac output. In KI mice, %FS remained significantly higher than in WT mice, and persisted up to 48 weeks. Collectively, the structural and functional characteristics observed in KI mice demonstrate progressive concentric left ventricular hypertrophy with severe reduction of left ventricular volume.

### KI mice demonstrate a decline in locomotor ability

**Fig. 4.**
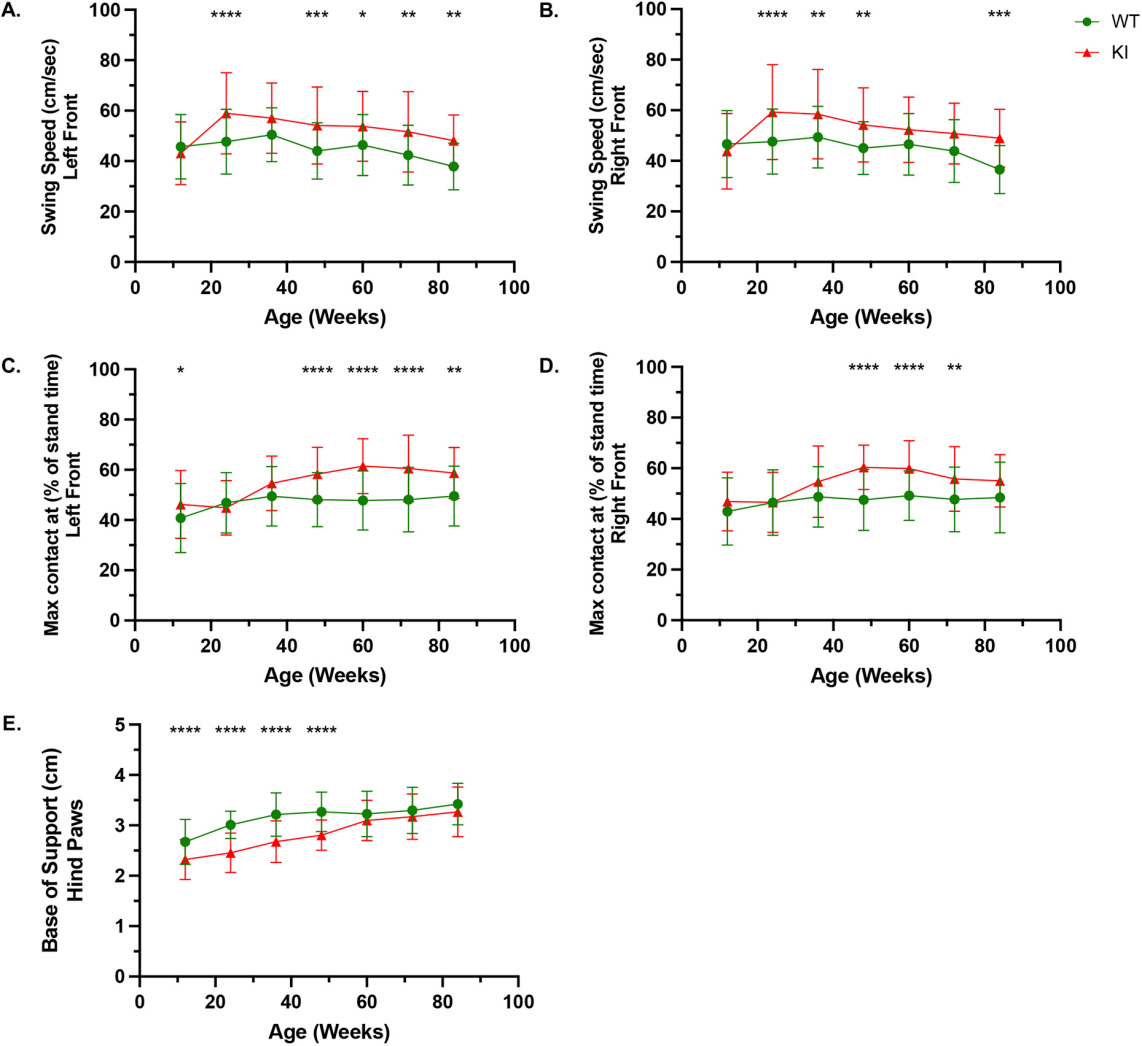
**KI mice exhibit deficits in locomotor function.** (A-E) KI mice exhibit a decline in locomotor function, as assessed by gait analysis of forelimb swing speed (A,B), maximum contact time (expressed as a percentage of stance time) in the forelimbs (C,D) and base of support in the hindpaws (E). Sample sizes (WT/KI): 12 weeks: *n*=24/27; 24 weeks: *n*=18/18; 36 weeks: *n*=18/18; 48 weeks: *n*=21/18; 60 weeks: *n*=17/18; 72 weeks: *n*=16/17; 84 weeks: *n*=16/14. Data were obtained from at least three independent experiments and shown as mean±s.d. All comparisons were analyzed using two-way ANOVA with Šídák post-hoc test. *P<0.05, **P<0.01, ***P<0.001, ****P<0.0001.

To assess locomotor behavior in mice, we evaluated gait by using CatWalk XT, a system that provides an objective, automated analysis of both dynamic and static aspects of locomotion. These aspects of locomotion are classified as four main categories: (1) run characteristics and kinetic parameters, (2) temporal parameters, (3) interlimb coordination parameters and (4) spatial parameters (Table S3). Among the key parameters analyzed, most were comparable between WT and KI mice, including average speed, cadence, number of steps, body speed, stand time, swing time, step cycle, stride length, base of support and print area (Figs S4-S7). Notably, three parameters, swing speed (Fig. 4A,B; Fig. S4H-K), maximum contact time (expressed as a percentage of stance time) (Fig. 4C,D; Fig. S5M-P) and base of support (Fig. 4E; Fig. S6E,F), showed significant differences between the two cohorts, highlighting specific gait alterations in KI mice. Analysis of the swing speed, defined as the velocity of a limb during the swing phase of the gait cycle, revealed a significant difference observed only in the forelimbs, where KI mice exhibited an increase in swing speed compared to WT mice beginning from 24 weeks (Fig. 4A,B). Analysis of the maximum contact time (expressed as a percentage of stance time), which represents the point during the stance phase of the gait cycle when the paw exerts the greatest pressure on the ground relative to the total duration of the stance phase, revealed a significant difference between WT and KI mice (Fig. 4C,D). These differences were only observed during the forelimb assessments, where KI mice exhibited a prolonged maximum contact time compared to WT controls. Base of support, measured as the distance between forepaws or hindpaws during locomotion, revealed a significant difference in the hindpaws only (Fig. 4E). WT mice exhibited a wider hindlimb base of support compared to KI mice during the first year of observation (12-48 weeks), while no significant differences were detected at later timepoints (60-84 weeks).

### KI mice exhibit physiological deterioration indicated by progressive attrition of muscular strength, decline in musculoskeletal coordination and reduction in body mass

**Fig. 5.**
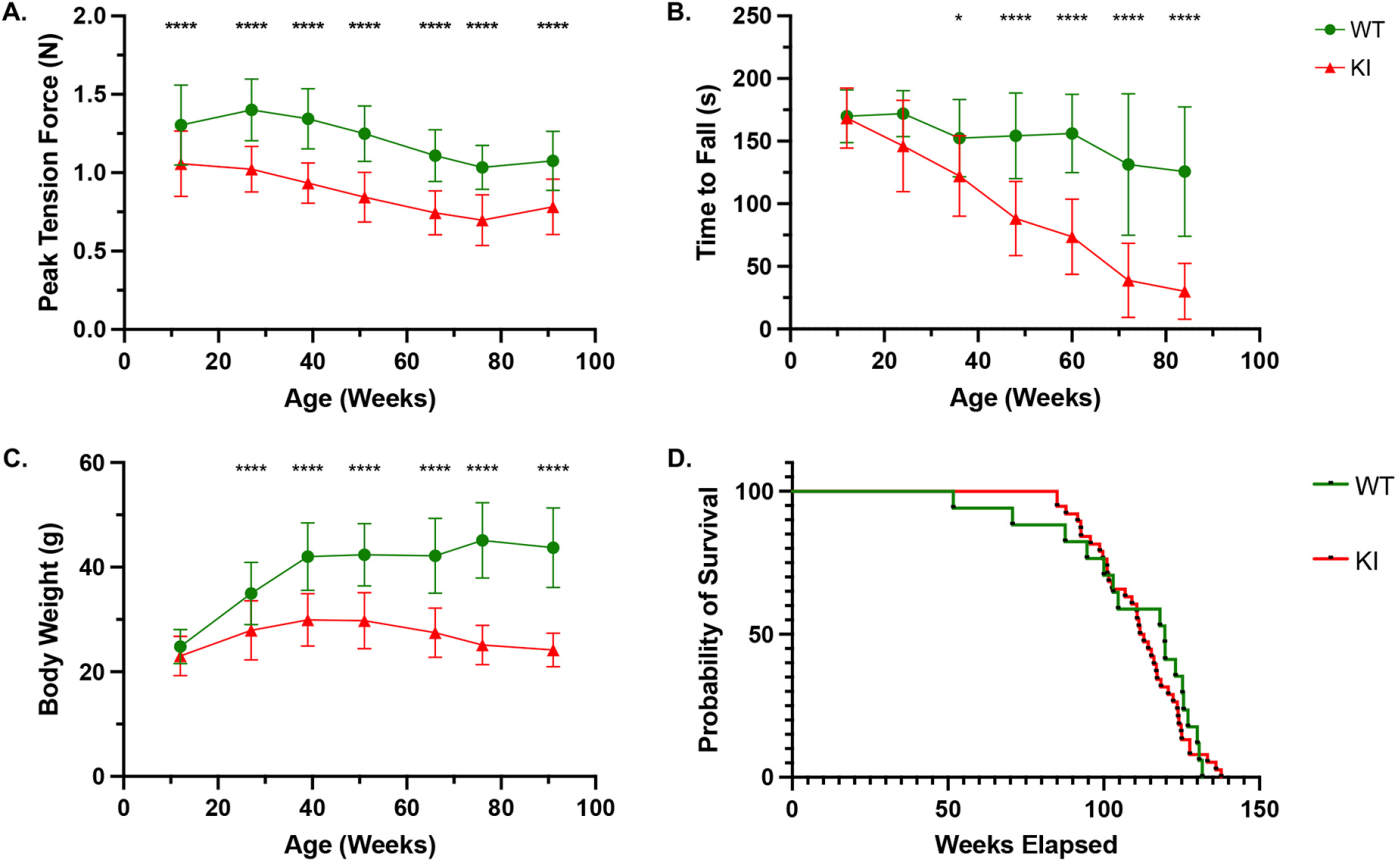
**KI mice exhibit reductions in muscular strength, motor coordination and body weight.** (A) KI mice exhibit reduced forelimb grip-strength force relative to WT mice from 12 weeks onwards, with progressive decline throughout their lifespan. Sample sizes (WT/KI): 12 weeks: *n*=87/90; 27 weeks: *n*=66/69; 39 weeks: *n*=63/72; 51 weeks: *n*=54/63; 66 weeks: *n*=45/51; 76 weeks: *n*=42/54; 91 weeks: *n*=27/45. (B) KI mice exhibit a progressive decline in motor coordination, as assessed by rotarod performance. Sample sizes (WT/KI): 12 weeks: *n*=18/29; 24 weeks: *n*=18/18; 36 weeks: *n*=18/18; 48 weeks: *n*=18/18; 60 weeks: *n*=17/18; 72 weeks: *n*=16/18; 84 weeks: *n*=16/18. (C) Significant reduction in body weight is evident in KI mice by 24 weeks of age, compared to WT mice. KI mice achieve peak body mass at 36 weeks, whereas WT mice reach their peak by 72 weeks. Sample sizes (WT/KI): 12 weeks: *n*=29/29; 27 weeks: *n*=23/23; 39 weeks: *n*=21/24; 51 weeks: *n*=18/21; 66 weeks: *n*=15/17; 76 weeks: *n*=14/18; 91 weeks: *n*=9/15. Data were obtained from at least three independent experiments and are shown as the mean±s.d. Body weight, grip strength, and rotarod comparisons were analyzed using two-way ANOVA with Šídák post-hoc test. *P<0.05, ****P<0.0001. (D) Kaplan–Meier survival curve indicating comparable survival rates between the study cohorts. Sample sizes (WT: *n*=19; KI: *n*=38). Survival was analyzed using log-rank (Mantel-Cox) test.

We have previously reported a significant reduction in forelimb peak tension force (N) of KI mice at 12 weeks of age (Huang et al., 2020). Grip-strength analysis presented in this current study further supports the progressive decline in muscular strength between 12 and 84 weeks of age. KI mice demonstrated decreased peak grip force of the forelimbs compared to that of WT mice (Fig. 5A).

To further investigate the impact of PD on musculoskeletal coordination and balance, rotarod performance was assessed for WT and KI mice at 12-week intervals. Differences in motor function became apparent, starting at 36 weeks, as KI mice demonstrated impaired motor performance with a significantly shorter latency to fall compared to WT controls. This trend persisted as the mice aged, with significant differences observed up to 84 weeks of age (Fig. 5B).

To monitor the general health of KI mice, body weight was assessed at 12-week intervals between 12 and 84 weeks of age. A significant reduction in body weight was observed in KI mice compared to WT mice, starting at 24 weeks (Fig. 5C).

### Survival analysis of WT and KI mice

Survival rates were assessed in WT and KI mice to evaluate the impact of the c.1826dupA mutation on longevity. Kaplan–Meier estimator of survival analysis revealed that KI mice exhibit a survival curve comparable to that of WT controls. The median survival was 119.7 weeks for WT mice and 112.3 weeks for KI mice (*n*=18 and 38, respectively), with no statistically significant difference in mortality between the groups (Fig. 5D).

### Reduced GAA activity and elevated glycogen content in KI compared to WT mice

To validate the biochemical characteristics of KI mice, GAA enzyme activity levels were analyzed in the heart, diaphragm, gastrocnemius and brain. The results demonstrated a sustained reduction in GAA activity in KI mice compared to WT mice throughout the duration of the study. Specifically, GAA activity was reduced by 91.6–96.6% in the heart, 92.1–93.0% in the diaphragm, 92.1–93.0% in the gastrocnemius and 85.6–95.6% in the brain of KI mice relative to their respective levels in WT mice (Table 1).

**Table 1.** Impaired GAA enzyme activity in KI mice

GAA enzyme activity (U/mg protein)					
Tissue	Genotype	12 weeks	24 weeks	60 weeks	72 weeks
HRT	WT	9.94±3.04	27.6±4.13	13.7±0.81	12.2±1.02
	KI	0.73±0.32****	0.95±0.12****	0.90±0.32****	1.02±0.27****
DIA	WT	8.82±0.80	8.51±0.69	9.81±1.04	9.82±2.55
	KI	0.62±0.06****	0.67±0.10****	0.69±0.14****	0.75±0.15****
GAS	WT	5.08±2.44	7.93±0.58	12.6±3.78	7.21±2.07
	KI	0.73±0.10***	0.49±0.09****	0.55±0.13****	0.54±0.10****
BRN	WT	56.6±28.3	94.0±10.2	89.8±8.27	79.9±8.15
	KI	0.98±0.07***	0.47±0.10****	0.72±0.17****	0.53±0.16****

GAA enzyme activity levels are significantly reduced in heart (HRT), diaphragm (DIA), gastrocnemius (GAS) and brain (BRN) of KI mice compared to WT controls at all timepoints assessed. One activity unit (U) was defined as 1 nmol substrate converted per hour. Sample sizes (WT/KI): 12 weeks: *n*=8/8; 24 weeks: *n*=6/6; 60 weeks: *n*=6/6; 72 weeks: *n*=6/6. Data were obtained from at least three independent experiments and shown as mean±s.d. Statistical comparisons for each tissue and corresponding timepoint were performed using two-tailed unpaired *t*-tests. ***P<0.001, ****P<0.0001.

We further assessed glycogen content levels in the same tissues and found a substantial increase in glycogen load in KI compared to WT mice. Specifically, glycogen levels were elevated up to 350-fold in the heart, 325-fold in the diaphragm, 30-fold in the gastrocnemius and 385-fold in the brain relative to those in WT mice; tissue glycogen content generally increased in KI mice with increasing age while remaining essentially constant in WT mice (Table 2).

**Table 2.** Glycogen accumulation in cardiac and skeletal muscles of KI mice

Glycogen content (μg/mg protein)					
Tissue	Genotype	12 weeks	24 weeks	60 weeks	72 weeks
HRT	WT	0.65±1.20	1.67±1.34	0.53±1.31	0.27±0.47
	KI	221±40.7****	184±38.9****	114±35.4****	94.4±61.8**
DIA	WT	0.93±1.79	0.27±0.65	3.23±3.35	1.56±1.95
	KI	63.1±15.6****	87.7±10.2****	123±10.3****	113±11.2****
GAS	WT	1.58±1.07	1.97±2.53	2.05±1.85	5.08±3.28
	KI	47.6±15.6****	51.0±10.3****	42.8±18.6***	76.2±21.7****
BRN	WT	0.10±0.29	0.42±1.02	0.18±0.34	0.13±0.31
	KI	25.0±5.71****	20.7±13.8**	63.9±10.5****	50.0±7.22****

Glycogen levels were significantly increased in heart (HRT), diaphragm (DIA), gastrocnemius (GAS) and brain (BRN) of KI mice compared to WT controls at all timepoints assessed. Sample sizes (WT/KI): 12 weeks: *n*=8/8; 24 weeks: *n*=6/6; 60 weeks: *n*=6/6; 72 weeks: *n*=6/6. Data were obtained from at least three independent experiments and shown as the mean±s.d. Statistical comparisons for each tissue and corresponding timepoint were performed using two-tailed unpaired *t*-tests. All comparisons were analyzed using two-tailed unpaired *t*-test. Significance levels are indicated as follows: **P<0.01, ***P<0.001, ****P<0.0001.

### Histopathological evaluation demonstrates increased PAS staining intensity in KI compared to WT mice

**Fig. 6.**
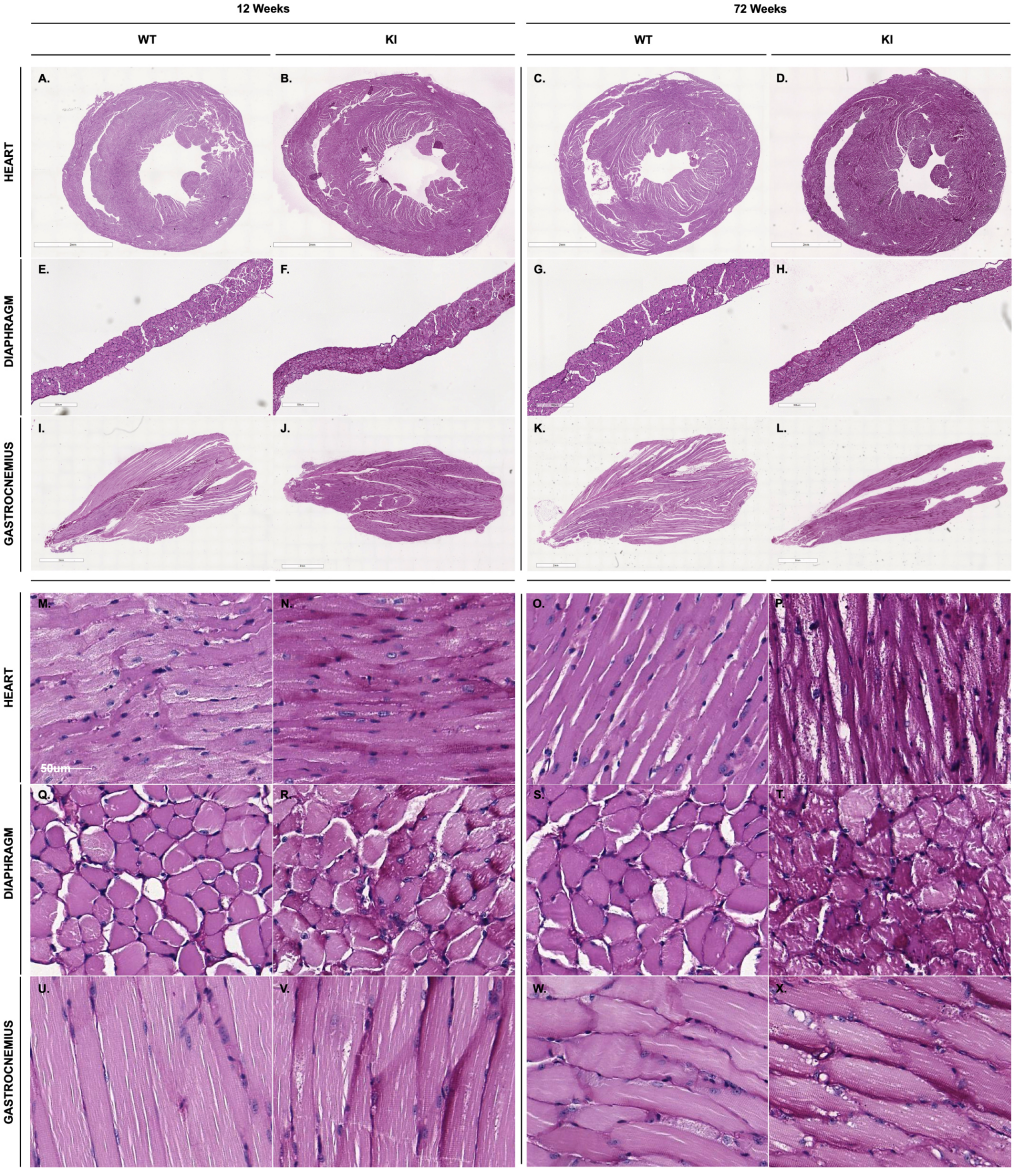
**Qualitative differences in PAS staining intensity between WT and KI tissues.** (A-X) Representative histological images of heart (A-D, M-P), diaphragm (E-H, Q-T) and gastrocnemius tissues (I-L, U-X) at 12 weeks (left) and 72 weeks (right) reveal increased PAS staining intensity in KI mice compared to WT controls. Notable differences in staining intensity are in the heart and gastrocnemius tissues, with more-subtle changes observed in the diaphragm.

To visualize and assess differences in glycogen accumulation, we utilized PAS staining of skeletal and cardiac muscle from WT and KI mice. Qualitatively, PAS staining appears more intense in KI mice, marked by a deeper magenta coloration in their tissues compared to that in WT mice. Representative images of PAS staining highlight varying degrees of staining intensity in the heart, diaphragm and gastrocnemius tissues at 12 weeks and 72 weeks timepoints (Fig. 6).

## DISCUSSION

This study provides one of the longest longitudinal characterizations of a murine PD model to date, highlighting the value of extended observation in uncovering the natural history of disease progression in preclinical models. Using this KI murine model, we systematically tracked disease progression over 84 weeks through physiological, behavioral, biochemical and histopathological analyses. This extended timeline provides profound insights into the natural course of PD, including early-onset HCM, progressive locomotor and skeletal muscle impairments, and significant lysosomal glycogen accumulation.

Cardiac involvement is characteristic of PD, marked by thickening of the left ventricular (LV) walls, reduction of LVID and progressive cardiac systolic dysfunction (Kodde et al., 2007). Our KI model demonstrates sustained cardiac dysfunction across all parameters assessed throughout the observation period (Figs 2 and 3). Most notable was progressive HCM, the defining pathological feature of IOPD, beginning at 12 weeks of age. This aligned with glycogen accumulation in cardiac myocytes, a hallmark of PD pathology, which can disrupt normal cellular function and trigger maladaptive hypertrophic responses (Kishnani and Howell, 2004). Consistent with this, our biochemical and histological analyses showed marked glycogen accumulation in cardiac tissues (Fig. 6, Table 2). In individuals with untreated IOPD, HCM is one of the primary determinants of disease progression. Without early intervention, affected individuals are at high risk of developing congestive heart failure and, ultimately, death (van Capelle et al., 2018). Together, these structural and functional changes to the heart contribute significantly to the overall pathology of human PD, impairing cardiac performance and playing a critical role in disease progression and mortality. While the cardiac abnormalities of the murine KI model parallel key pathological features observed in human IOPD, they do not progress to overt heart failure or early mortality in this model.

Progressive compromise in musculoskeletal integrity, encompassing fine and gross motor functions such as strength, coordination, balance and postural stability, is also a hallmark of PD (Manganelli and Ruggiero, 2013; McIntosh et al., 2015; Watkins et al., 2023). Deterioration of skeletal muscle function is the result of glycogen accumulation, which disrupts sarcomere architecture and impairs contractility (Schänzer et al., 2017). This, coupled with reported neurological involvement in PD, further exacerbates the impact on normal motor function and control (Giselbrecht et al., 2019). These outcomes can be observed in other animal models of PD, including zebrafish, quail, rat and one of the most-characterized animal models of PD to date, the B6;129-*Gaa^tm1Rabn^*/J mouse (Bragato et al., 2020; Kikuchi et al., 1998; Muñoz et al., 2024; Sidman et al., 2008). Comparatively, we observed similar outcomes in our KI mouse model, with a decline in muscular strength (Fig. 5A), impaired motor coordination and balance (Fig. 5B), and deficits in locomotion (Fig. 4; Figs S4-S7).

A decline in body mass, commonly observed in PD, highlights the disease-associated progressive muscle atrophy and reduced physical function. Impaired feeding abilities are characteristic of individuals with PD, often leading to insufficient nutrition and growth (Kishnani and Howell, 2004). In older KI mice, PD-induced muscle weakness hinders their ability to access food and water in a standard cage setup. Consequently, food and water must be positioned within an accessible range to ensure proper care. From 24 weeks of age, KI mice exhibited a substantial reduction in body mass compared to WT mice. On average, KI mice maintained a body mass of 20-30 g, whereas WT mice sustained a higher average mass of 40-50 g (Fig. 5C). In PD, disruption of the cellular mechanisms responsible for glycogen processing leads to the accumulation of glycogen within lysosomes, particularly in skeletal muscle. Over time, the progressive glycogen burden induces lysosomal rupture, triggering muscle cell death. This cascade ultimately results in muscle wasting and a significant loss of muscle mass, contributing to a decline in overall body weight (Hesselink et al., 2003; van den Berg et al., 2013). Physical activity is known to mitigate muscle mass loss and counteract muscle aging (Distefano and Goodpaster, 2018). However, the ability to engage in physical activity is compromised in neuromuscular diseases, the severity varying by disease. This impairment hinders muscle strength maintenance, accelerating muscle degeneration (McDonald, 2002). Given these progressive effects on muscle and cardiac tissues, direct measurement of individual organ weights would have provided additional insight into atrophy and cardiomegaly. A limitation of this study is the absence of such measurements. Nonetheless, our comprehensive physiological and functional assessments – including echocardiography, longitudinal body weight and behavioral testing – offer robust indicators of these disease manifestations. Future studies may incorporate direct organ-weight measurements.

In patients, the reduction in GAA enzyme activity across various tissues underscores the enzymatic deficiency characteristic of PD. GAA deficiency leads to intra-lysosomal glycogen storage that profoundly affects cardiac and skeletal muscle cells due to their high metabolic demands (Byrne et al., 2011; Kodde et al., 2007; Periasamy et al., 2017). In this study, biochemical analyses revealed decreased GAA activity with concordant elevation of lysosomal glycogen storage in the heart, diaphragm, gastrocnemius and brain of KI mice (Tables 1 and 2). Our findings align with the pathological features observed in humans with IOPD, highlighting muscle-specific vulnerability that manifests in symptomatic deficits, such as HCM, respiratory insufficiency and myopathy. Moreover, these results exhibit a biochemical resemblance to the previously characterized KO murine model of PD, the B6;129-*Gaa^tm1Rabn^*/J mouse (Raben et al., 1998). We observed a time-dependent decrease in glycogen content in the heart tissues of KI mice, a pattern also seen in the KO model but with some differences regarding the rate of progression. This unexpected finding may be attributed to the progressive loss of cardiomyocytes. We hypothesize that, as cells undergo death and are replaced by fibrotic tissue, the altered metabolic profile of the fibrotic tissue – which cannot maintain normal metabolic functions – disrupts glycogen storage in cardiomyocytes. Previous studies have shown that fibrotic tissue exhibits irregular metabolism in both liver and pulmonary fibroses, suggesting that fibrosis disrupts normal metabolic processes in affected tissues (Delgado et al., 2021; Rajesh et al., 2023). Consequently, this replacement leads to a reduction in measurable glycogen content. Histopathological findings in *Gaa* c.1826dupA KI mouse muscle support these biochemical data, providing qualitative evidence of glycogen accumulation. Intensified PAS staining was observed in KI tissues, particularly in the heart and gastrocnemius (Fig. 6). Together with direct biochemical measurements of glycogen content, these findings highlight tissue-specific patterns of glycogen storage. While our analyses demonstrate glycogen accumulation and reduced GAA activity in the brain, we did not further characterize CNS pathology or neurological function, limiting conclusions regarding neurodegeneration or CNS-mediated functional impairment. This limited scope represents a study limitation and highlights the need for future work to define CNS involvement more comprehensively in this model. Nevertheless, the presence of glycogen accumulation in the brain highlights the utility of this model for future studies examining CNS involvement and therapeutic resistance.

KI mice experience no significant change in lifespan, thereby deviating from the typical disease-course in untreated humans with IOPD. While untreated human patients with IOPD typically succumb to the disease within the first year of life, equivalent to ∼9 days of age in mice, our KI mice live well into adulthood, showing a lifespan of up to 2.5 years, comparable to that of WT mice (Fig. 5D) (Dutta and Sengupta, 2016). This prolonged survival suggests the existence of compensatory or adaptive mechanisms that mitigate disease severity in mice despite substantial biochemical pathology. The underlying mechanism for this disparity remains unknown but has also been observed in the B6;129-*Gaa^tm1Rabn^*/J mice (Raben et al., 1998). Variations in survival may be attributed to inherent physiological differences between humans and mice, presenting a significant limitation when directly extrapolating outcomes in murine models to those in humans, particularly in the context of therapeutic interventions. Further investigation is needed to elucidate the underlying causes of these differences, potentially through gene-expression profiling using RNA sequencing (RNAseq) or other analytical approaches.

The phenotypes observed in our KI murine model capture key features of human IOPD, including early and sustained HCM, progressive skeletal muscle weakness, impaired motor function, and widespread glycogen accumulation. However, it is important to note that this model does not reproduce several hallmark clinical outcomes of severe IOPD, including early lethality, failure to thrive, overt cardiac failure and respiratory insufficiency. These limitations underscore species-specific differences in disease progression and physiological compensation. Despite these differences, the model exhibits a reproducible trajectory of pathology that enables the longitudinal evaluation of therapeutic strategies at the mechanistic and preclinical level. At 12 weeks of age, animals display significant cardiac hypertrophy, reduced forelimb peak tension strength, marked tissue glycogen accumulation and severe acid α-glucosidase deficiency, providing robust and quantifiable endpoints for preclinical efficacy studies. Importantly, the relative survival of these mice in the absence of therapy may offer unique experimental opportunities. First, this model may facilitate the investigation of genetic, metabolic or environmental modifiers that exacerbate disease severity and accelerate functional decline. Second, the prolonged survival suggests the presence of adaptive or compensatory mechanisms that permit tissue function despite substantial glycogen burden, providing a platform to interrogate pathways that may modulate disease tolerance or resilience. Third, the model enables focused investigation of CNS involvement in PD, an area that remains challenging to address when using current enzyme-replacement or liver-directed gene therapies and may be relevant to predicting late-onset or therapy-modified phenotypes. Collectively, while the KI model does not fully reproduce the lethality or multisystem failure observed in human IOPD, it provides a stable and mechanistically informative system for studying disease progression, modifier biology and tissue-specific therapeutic responses.

To contextualize these findings, we present a comprehensive summary of previously reported murine models of PD (Table 3), which details prior mouse and rat PD models, their genetic modifications, and key limitations of each system. Many commonly used KO models, including the B6;129-*Gaa^tm1Rabn^*/J and related *Gaa*-null lines, exhibit complete absence of GAA activity, attenuated disease progression, strain-dependent phenotypic variability and lack of early lethality or respiratory failure, thereby, limiting their ability to model the spectrum and temporal evolution of human disease. Other engineered models introduce additional genetic modifications (e.g. autophagy suppression or immunodeficiency) that, while useful for specific mechanistic or therapeutic questions, alter core cellular pathways or immune responses and, thus, diverge from the native pathophysiology of PD. In contrast, the *Gaa^c^*^.1826dupA^ KI model retains the endogenous regulatory context while introducing a disease-relevant mutation, producing progressive cardiac and skeletal muscle pathology, sustained biochemical deficiency and functional decline without the confounding effects of complete gene ablation or pathway disruption. This genetic configuration enables the longitudinal assessment of disease trajectory, investigation of modifier mechanisms that influence severity and tissue vulnerability, and evaluation of therapeutic strategies –such as gene editing, allele-specific correction, or tissue-targeted delivery – under conditions that more closely reflect patient-relevant molecular defects. Accordingly, the *Gaa^c^*^.1826dupA^ model provides a complementary and mechanistically informative platform that bridges the gap between severe but genetically artificial KO systems and the complex mutation-driven pathology observed in human PD.

**Table 3.** Overview of PD rodent models

**Species**	**PD model name(s)**	**Genomic modification**	**Limitations**	**References**
Mouse	*Gaa*^−/−^ (*AGLU^−/−^,* GSDII KO mice)	KO of the murine *Gaa* gene causing complete loss of acid α-glucosidase (GAA) activity; a *neo* cassette was inserted into the unique *Eag*I restriction site within exon 13 of *Gaa* (Bijvoet et al., 1998).	KO of *Gaa* gene; complete loss of GAA activity may not reflect the variability and residual enzyme activity seen in patient phenotypes. Lack of overt clinical symptoms (muscle weakness, respiratory insufficiency, etc.); remain asymptomatic throughout early life. Does not exhibit early lethality as seen in IOPD.	Bijvoet et al., 1998
Mouse	B6;129-*Gaa^tm1Rabn^*/J (*Gaa*^−/−^, *Gaa*^6neo/6neo^)	KO of the murine *Gaa* gene causing complete loss of acid α-glucosidase (GAA) activity; a termination codon and a neomycin resistance cassette flanked by loxP sites were inserted into exon 6 of *Gaa* (Raben et al., 1998).	KO of *Gaa* gene; complete loss of GAA activity may not reflect the variability and residual enzyme activity seen in patient phenotypes. Attenuated pace of disease and clinical severity compared to adult/infantile forms of human disease. Phenotype influenced by genetic/strain background; a milder muscle phenotype in *GAA* KO in a mixed C57BL/6;129;FVB background compared to the most-severe *Gaa* KO^B6;129^ strain. Does not exhibit early lethality as seen in IOPD.	Raben et al., 1998
Mouse	C57BL/6NJ-*Gaa^em2Jhng^*/J (*Gaa*^c.1935C>A/c.1935C>A^)	KI of the human-relevant c.1935C>A (p.Asp645Glu) variant in murine *Gaa* gene; CRISPR/Cas9-mediated nucleotide substitution (C>A) introduced an Asp 645Glu amino acid change (Kan et al., 2022).	No respiratory involvement has been described to date. Natural history, survival and longitudinal disease progression are still under study. Despite near-null GAA activity, KI mice lack neonatal or early postnatal lethality; similar to most murine PD models it does not recapitulate this aspect of untreated human IOPD.	Kan et al., 2022
Mouse	Myl1^tm1(cre)Sjb^ Atg7^tm1Tchi^ Gaa^tm1Rabn^/PuroMmjax (MLCcre:Atg7^F/F^:GAA^−/−^)	KO of the murine *Gaa* gene combined with skeletal muscle–specific deletion of *Atg7* to suppress autophagy in muscle; conditional *Atg7* KO mice were crossed with *Gaa^−/−^* mice, and the resulting line was then crossed to a skeletal muscle–specific MLC-Cre line to generate *Gaa^−/−^* mice lacking *Atg7* specifically in skeletal muscle (Raben et al., 2010).	Muscle-specific autophagy deficiency may exaggerate or mask aspects of pathology Model restricted to skeletal muscle; does not reflect systemic PD (effect on heart, CNS, smooth muscle). Compensatory pathways (noncanonical autophagy) may confound results; suppressing *Atg7* can trigger non-canonical autophagy or other compensatory mechanisms, which may differ between mice and humans.	Raben et al., 2010
Mouse	HSA-*Cre*:*Atg5^flox/flox^*: *GAA*−/− (AD-GAA KO)	KO of the murine *Gaa* gene combined with skeletal muscle-specific deletion of *Atg5* to study pathology modulation; *Atg5* conditional KO mice were crossed with transgenic HSA-Cre mice to generate mice with muscle-specific *Atg5* deficiency. These mice were then crossed with *GAA* KO mice to produce *GAA* KO mice lacking *Atg5* specifically in skeletal muscle (Raben et al., 2008).	Global suppression of autophagy exaggerates pathology; complete *Atg5* deletion creates a more-severe, non-physiologic phenotype than typically observed in human PD. Confounding disease mechanisms: severe accumulation of ubiquitylated proteins and toxic inclusions due to loss of autophagy, making it difficult to isolate primary pathology of PD. Does not model the localized autophagic dysfunction seen in human PD patients; enforces uniform autophagy deficiency rather than fibre-restricted, incomplete autophagic flux characteristic of PD muscle.	Raben et al., 2008
Mouse	Tag(CAG-EGFP/Map1lc3b)53Nmz *Gaa^tm1Rabn^*/RabnMmjax (GFP:LC3-*GAA*-/-)	Transgenic expression of GFP-LC3 reporter on *Gaa* KO background for *in vivo* monitoring of autophagy/autophagosomes; GFP:LC3-GAA*^−/−^* mice carry a null allele (*neo* inserted into exon 6) of *Gaa* and express the EGFP reporter gene fused to *Map1lc3b* (Lim et al., 2018).	Primarily a mechanistic/reporter model rather than disease-severity model; GFP-LC3 tagging is designed to visualize autophagy and study protein turnover rather than replicate full clinical PD phenotypes. Focuses on catabolic signaling in skeletal muscle tissue (UPS, mTOR, autophagy) and does not comprehensively model cardiac, respiratory or CNS involvement. Therapeutic findings may be context dependent; strategies, such as autophagy suppression or mTOR activation, improve responsiveness to enzyme replacement therapy in this system but may introduce cellular stress, ubiquitylated protein accumulation or translational suppression that are not reflective of PD patient biology. Limited translational relevance for long-term therapy; chronic manipulation of proteasome or mTOR pathways has been acknowledged to carry potential metabolic and toxicity risks.	Lim et al., 2018
Mouse	*GAA*-KO:SCID	KO of the murine *Gaa* gene on a severe combined immunodeficient (SCID) background; *Gaa^−/−^* mice were crossed with SCID mice to generate *Gaa-*KO*/*SCID double-KO mice (Xu et al., 2004).	Immunodeficient background does not recapitulate human immune responses to GAA protein or gene therapy, masking clinically relevant immunogenicity effects (e.g. antibody formation, infusion reactions). Functional readouts may be artificially prolonged or enhanced (because immune responses that would normally limit therapy are absent), potentially overestimating therapeutic benefit.	Xu et al., 2004
Mouse	*Gaa* KO^DBA2/J^	KO of the murine *Gaa* gene on a DBA2/J background showing severe respiratory and spinal pathology; the *Gaa*^B6;129^ KO strain was crossed with DBA2/J mice to generate a novel *Gaa* strain homozygous for the *Ltbp4^Δ36^* allele (Colella et al., 2020).	Sex-specific effects; male mice show early lethality and more-severe respiratory defects, while females show milder phenotypes. Phenotype influenced by genetic/strain background; respiratory defects are pronounced due to the DBA2/J background and *Ltbp4^Δ36^* allele, which may limit generalizability. The current mixed genetic (C57BL/6;129;DBA2/J) background may introduce variability and confound comparisons.	Colella et al., 2020
Rat	*Gaa* (Rat) R385STOP (SD-Gaa^em1Fbos) (*Gaa*^−/−^)	KI of a nonsense mutation (p.Arg385*) in rat *Gaa* (catalytic site) causing severe PD with early-onset pathology; *Gaa^−/−^* rats carry the CRISPR/Cas9-mediated C>T single-nucleotide polymorphism and a *Bse*NI restriction site in exon 7 of *Gaa* (Muñoz et al., 2024).	Complete Gaa KO with no residual enzyme activity; may not reflect the spectrum of human Pompe phenotypes that retain partial GAA function, particularly in LOPD. Extremely rapid disease progression and short lifespan (<8 months); limits long-term natural history studies and evaluation of chronic therapeutic durability.	Muñoz et al., 2024

IOPD, infantile-onset Pompe disease; KI, knock-in; KO, knockout; LOPD, late-onset Pompe disease; PD, Pompe disease.

Building on these parallels, our findings highlight the relevance of the KI mouse model as a resource for investigating disease mechanisms and targeted therapeutic strategies. For individuals with PD, the current standard of care and only available therapeutic intervention is human acid α-glucosidase enzyme replacement therapy, which supplements the deficient enzyme but requires lifelong weekly intravenous infusions, thereby, presenting significant logistical and clinical challenges. Consequently, the characterization of the KI model presented here provides an important preclinical foundation for evaluating next-generation therapies, including gene editing approaches. This model enables rigorous preclinical assessment of novel therapies under conditions reflecting key pathological features, thereby, supporting the development of potentially life-saving interventions.

## MATERIALS AND METHODS

### Animal usage

All animal procedures were approved by the Children's Hospital of Orange County Institutional Animal Care and Use Committee (IACUC protocol #IACUC-24-021; CHOC IACUC protocol #160902) and conducted in accordance with the NIH Guide for the Care and Use of Laboratory Animals and ARRIVE guidelines.

G2 mice were backcrossed for ten generations onto a C57BL/6NJ background prior to characterization. Mice were housed in a temperature-controlled facility under a 12-h light/dark cycle, with *ad libitum* access to PicoLab^®^ Rodent Diet 20 (LabDiet, St. Louis, MO, USA) and water. Environmental enrichment included ALPHA-dri^®^ bedding, 2-ply tissue, Bio-tunnels and igloos. Mice were group-housed by gender (except for mating trios), and heterozygous (*Gaa^wt/c.1826dupA^*) males and females were crossed to generate homozygous KI and WT mice. All experiments were performed on age-matched littermates. Genotyping for the *Gaa* c.1826dupA allele was performed using PCR with primers flanking exon 13 (1826dupA-F: 5′-CACTCAGGGCCCTGGTCAAGA-3′ and 1826dupA-R: 5′-GTGCCACATGAGATGGCGCATG-3′), followed by *Rsa*I digestion to distinguish WT from mutant alleles (Fig. S8).

### Sex as a biological variable

All experimental cohorts included male and female mice, with balanced representation across groups. Statistical analyses incorporated sex as a biological variable to evaluate potential sex-dependent effects (Figs S1, S2, S3; Tables S1, S2). Aside from a difference observed in rotarod performance, no significant sex-related effects were identified across measured outcomes. Accordingly, data obtained from males and females were pooled for all subsequent analyses and presentation.

### Murine echocardiography

One day prior to scanning, fur in the thoracic region of the mice was removed using depilatory cream to ensure quality image acquisition during echocardiographic analysis. Lubricant ointment was applied to the eyes immediately preceding the scan to prevent scleral drying. Mice were then restrained, placed on a heated platform and anesthetized with 5% isoflurane via a nose cone for 10-15 s, and maintained at 0.5% isoflurane throughout the examination. Electrodes for simultaneous electrocardiography were inserted into the upper and lower thoracic areas to monitor the heart rate. Transthoracic echocardiography, including M-mode (one-dimensional) and B-mode (two-dimensional), was performed using the Vevo 3100 high-resolution ultrasound system with a 25–55 MHz linear transducer (FUJIFILM VisualSonics Inc., Toronto, Canada). All measurements of heart function and structure were taken while maintaining a heart rate of 500 beats per minute (bpm) or higher.

Acquired images were then imported into the Vevo LAB ultrasound analysis software (FUJIFILM VisualSonics Inc.), from which the sonographer was able to measure multiple cardiac metrics, such as the left ventricular internal diameter (LVID), and thickness of the interventricular septum (IVS) and left ventricular posterior wall (LVPW), throughout systolic and diastolic phases. All reported values represent the means of at least three independent measurements. The left ventricular mass index (LVMI) and percentage of fractional shortening (%FS) were calculated as previously described (Huang et al., 2020).

### Locomotor behavior

Gait analysis was conducted utilizing the CatWalk XT system (Noldus Information Technology, Leesburg, VA, USA) according to the manufacturer's guidelines. Run criteria for image capture were defined prior to measurement and included minimum run duration of 0.50 s, a maximum of 12.30 s and at least three compliant runs. Any run outside these parameters was excluded. Experimental detection settings are as follows: camera gain (22 dB), green light intensity threshold (0.10), red ceiling light (17.7 V) and green walkway light (16.5 V). Mice were acclimated in a dark testing room for at least 1 h prior to measurement. Run criteria and walkway calibration were completed before starting each experiment, while background images of the empty walkway were captured before each trial. One trial consisted of three consecutive runs, with one run defined as a single translocation from one end of the walkway to the other. Mice entered from a single point and were recorded from below. Each run was processed using CatWalk XT software (Noldus Information Technology), which autogenerated raw data. Over 200 parameters were collected and organized into four primary categories (Table S3).

### Forelimb grip-strength assay

Muscle strength was evaluated using a forelimb grip-strength test, which measures peak tension force (in N) to assess neuromuscular function. Mice were acclimated at least 1 h prior to testing. Forelimb grip strength was measured using a horizontally oriented grip strength meter (Columbus Instruments, Columbus, OH, USA), consisting of a pull bar attached to a digital force transducer. Mice were guided by the tail to grasp the bar with both forepaws, then gently pulled back to elicit peak force. A valid measurement required full forelimb grip, steady resistance without premature release or turning and consistent pulling motion by the operator.

### Motor coordination and balance

Neuromuscular coordination and postural control were evaluated using a Rota-Rod apparatus (Med Associates Inc, Fairfax, VT, USA). Mice were placed on a horizontally rotating rod and required to ambulate forwards to avoid falling. Performance was measured by latency to fall (in seconds). The assessment was carried out over two days: Day 1 included a practice session with two 45-s trial rounds at a rotation speed of four rotations per minute (rpm), separated by a 5-min rest. Day 2 consisted of three test trials, each lasting up to 180 s with speed increasing from four to 25 rpm. The average latency to fall was calculated per mouse.

### Tissue harvesting

Mice were sacrificed by asphyxiation of CO_2_ at 12, 24, 60 and 72 weeks for biochemical and histological analyses (see Fig. 1) Following cardiac perfusion with cold phosphate-buffered saline, the heart, diaphragm, gastrocnemius muscles and brain were harvested. Each organ was bisected, with one half rapidly frozen and stored at −80°C for subsequent biochemical analysis, and the other fixed in ZinFix^®^ (E K Industries Inc, Joliet, IL, USA) for 48 h in preparation for histopathological processing.

### Biochemical analyses

GAA enzymatic activity and tissue glycogen content were assessed as previously described (Huang et al., 2020). Briefly, mouse tissues were homogenized in Cell-Lytic-M reagent (MilliporeSigma, Burlington, MA, USA), and GAA activity was measured using 4-methylumbelliferyl-α-d-glucopyranoside substrate in Mcilvaine buffer (citrate/phosphate buffer, pH 4.3), with fluorescence detected at 360/450 nm. One activity unit (U) was defined as 1 nmol substrate converted per hour and normalized to protein concentration determined by BCA assay (Thermo Fisher Scientific, Waltham, MA, USA). Specific activity reported in the study was defined as U per mg of protein (U/mg protein). Glycogen content was quantified using a commercial glycogen assay kit (Sigma-Aldrich, St. Louis, MO, USA), with absorbance measured at 570 nm and background glucose levels corrected by using non-hydrolyzed controls. Results were recorded as glycogen/ protein [µg/mg].

### Tissue processing and histological staining

Tissue processing and histological staining were carried out by the UCI Department of Pathology – Experimental Tissue Resource. Organs were embedded in paraffin, sectioned at 4 μm, and prepared as transverse sections for heart and diaphragm tissues, and longitudinal sections for gastrocnemius muscle. PAS and PAS–diastase (PAS-D) staining were conducted following histology core protocols. Representative images were captured at 20× magnification using the SL5 Real-Time telemicroscopy imaging system (Mikroscan Technologies, Carlsbad, CA, USA).

### Statistics

Statistical analyses were performed using GraphPad Prism (version 10.1). All behavioral experiments were conducted with a minimum of nine animals per group (*n*=9) to ensure statistical validity. Data are presented as the mean±standard deviation (±s.d.) unless otherwise indicated. Comparisons between two groups at a single timepoint were made using unpaired two-tailed Student's *t*-test. For comparisons involving two groups across multiple timepoints, one-way or two-way ANOVA followed by appropriate post hoc tests (e.g. Tukey's or Šídák's multiple comparisons) was used. Survival analysis was conducted using Kaplan–Meier curves and log-rank (Mantel–Cox) tests, with *P*<0.05 considered to be statistically significant.

## Supplementary Material

10.1242/dmm.052611_sup1 Supplementary information
