# Supplementary material for: Longitudinal characterization of Gaac.1826dupA mice reveals the cardiac, myopathic and biochemical phenotypes of Pompe disease
Source: Dis Model Mech. 2026 Mar 18;19(3):dmm052611. doi: 10.1242/dmm.052611 (PMC13035063; doi:10.1242/dmm.052611)
Supplement: Supplementary information [file dmm-19-052611-s1.pdf]

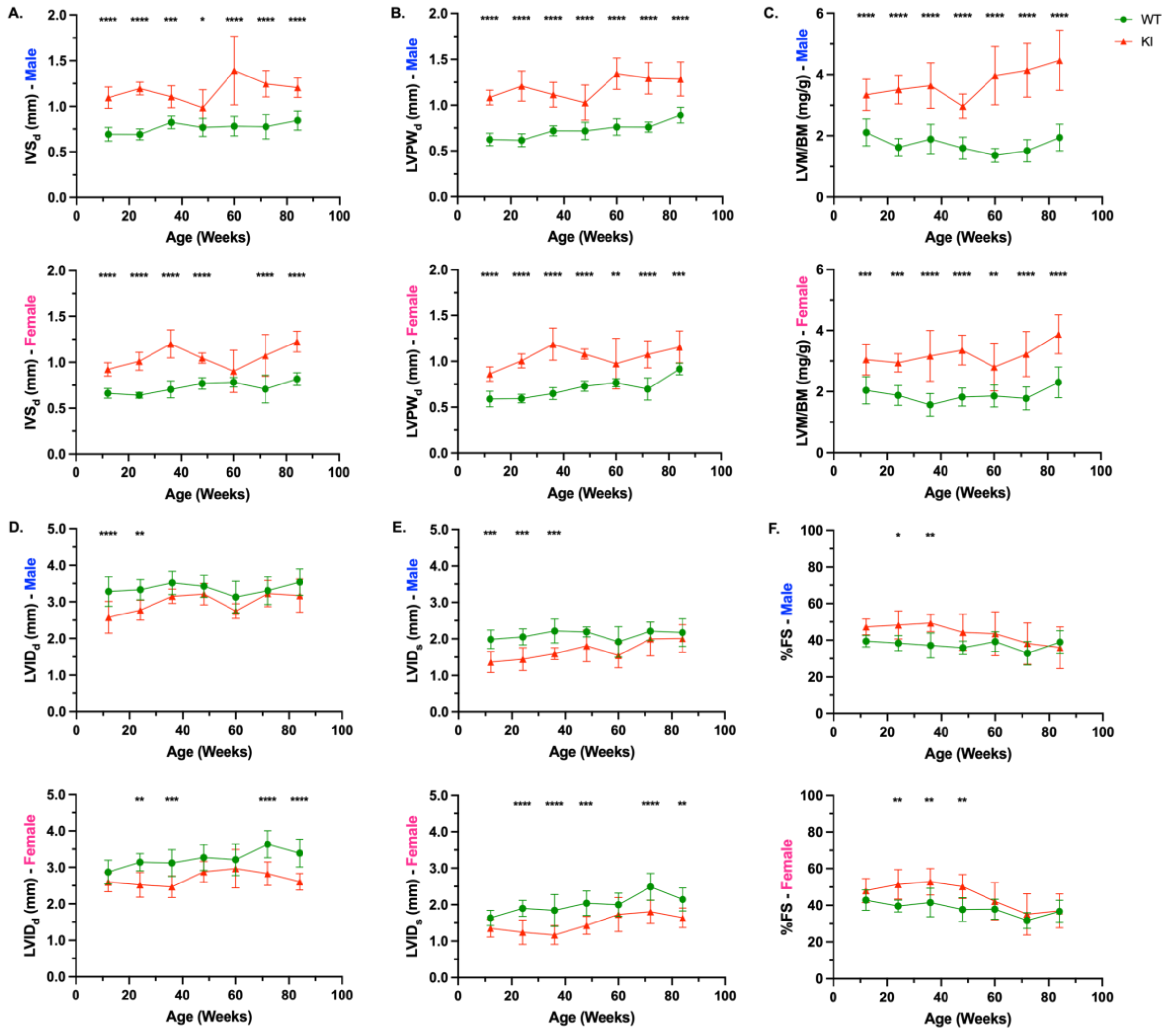

Echocardiography MALE	Timepoint	12wk	24wk	36wk	48wk	60wk	72wk	84wk
	WT (n=)	11	9	9	9	8	8	7
	KI (n=)	10	9	9	9	9	9	9

Echocardiography FEMALE	Timepoint	12wk	24wk	36wk	48wk	60wk	72wk	84wk
	WT (n=)	9	9	9	9	9	9	9
	KI (n=)	11	9	9	9	9	9	9

Fig. S1. Comprehensive cardiac evaluation of WT and KI mice. Comparative analysis of **(A)** IVS$_d$ **(B)** LVPW$_d$ **(C)** LVMI: LVM/BW **(D)** LVID$_d$ **(E)** LVID$_s$ **(F)** %FS reveals progressive deterioration of cardiac parameters in KI mice over 84 wks. Data were generated from at least three independent experiments and shown as mean ± s.d. All comparisons were analyzed using two-way ANOVA with Šídák post-hoc test. Significance levels are indicated as follows: *$p < 0.05$, **$p < 0.01$, ***$p < 0.001$, ****$p < 0.0001$. Analyses stratified by sex yielded comparable results, with no evidence of sex-specific effects.

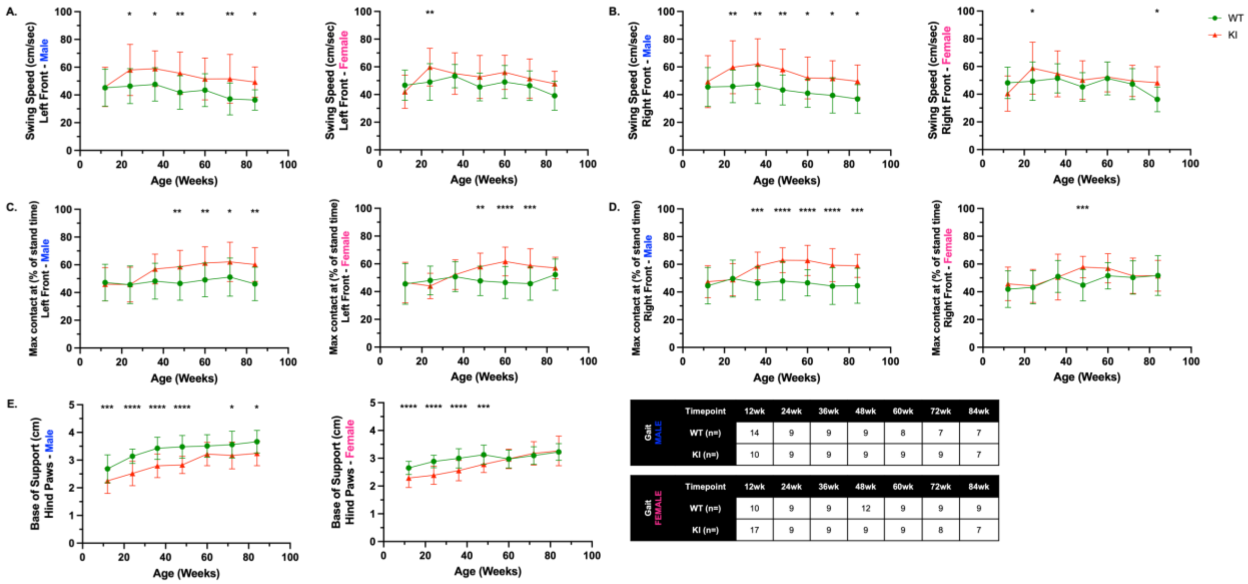

Gait MALE	Timepoint	12wk	24wk	36wk	48wk	60wk	72wk	84wk
	WT (n=)	14	9	9	9	8	7	7
	KI (n=)	10	9	9	9	9	9	7

Gait FEMALE	Timepoint	12wk	24wk	36wk	48wk	60wk	72wk	84wk
	WT (n=)	10	9	9	12	9	9	9
	KI (n=)	17	9	9	9	9	8	7

Fig. S2. KI mice exhibit deficits in locomotor function. KI mice exhibit a decline in locomotor function, as assessed by **(A-B)** gait analysis of forelimb swing speed, and **(C-D)** gait analysis of maximum contact time (expressed as a percentage of stance time) in the forelimbs, and **(E)** gait analysis of base of support in the hindpaws. Data were generated from at least three independent experiments and shown as mean ± s.d. All comparisons were analyzed using two-way ANOVA with Šídák post-hoc test. Significance levels are indicated as follows: *p<0.05, **p<0.01, ***p<0.001, ****p<0.0001. Male and female cohorts exhibited similar responses, with no sex-dependent variation observed.

Disease Models & Mechanisms • Supplementary information

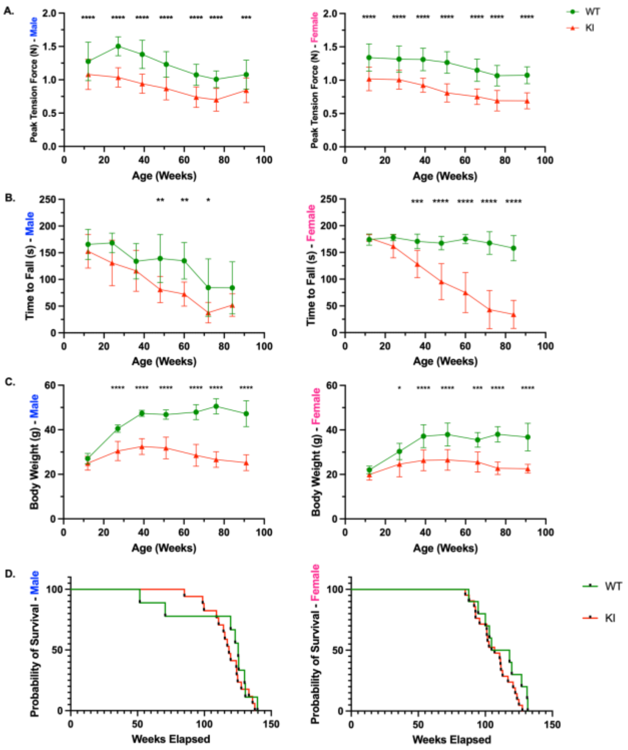

Grip Strength MALE	Timepoint	12wk	27wk	39wk	51wk	66wk	76wk	91wk
	WT (n=)	48	30	30	27	24	24	18
	KI (n=)	54	39	42	36	33	33	27

Grip Strength FEMALE	Timepoint	12wk	27wk	39wk	51wk	66wk	76wk	91wk
	WT (n=)	39	36	33	27	21	18	9
	KI (n=)	36	30	30	27	18	21	18

Rotarod MALE	Timepoint	12wk	24wk	36wk	48wk	60wk	72wk	84wk
	WT (n=)	9	9	9	8	8	7	7
	KI (n=)	11	9	9	9	9	9	9

Rotarod FEMALE	Timepoint	12wk	24wk	36wk	48wk	60wk	72wk	84wk
	WT (n=)	9	9	9	9	9	9	9
	KI (n=)	18	9	9	9	9	9	9

Weight MALE	Timepoint	12wk	27wk	39wk	51wk	66wk	76wk	91wk
	WT (n=)	16	11	10	9	8	8	6
	KI (n=)	18	13	14	12	11	11	9

Weight FEMALE	Timepoint	12wk	27wk	39wk	51wk	66wk	76wk	91wk
	WT (n=)	13	12	11	9	7	6	3
	KI (n=)	11	10	10	9	6	7	6

Survival MALE		
	WT (n=)	9
	KI (n=)	17

Survival FEMALE		
	WT (n=)	10
	KI (n=)	21

Fig. S3. KI mice exhibit reductions in muscular strength, motor coordination and body weight. (A) KI mice exhibit reduced forelimb grip strength force relative to WT mice from 12 weeks with a progressive decline throughout their lifespan. **(B)** KI mice exhibit a progressive decline in motor coordination, as assessed by rotarod performance. WT males demonstrated progressively lower performance compared to females beyond 24 wks. **(C)** A significant reduction in body weight is evident in KI mice by 24 wks of age, compared to WT mice. KI mice achieve peak body mass at 36 wks, whereas WT mice reach their peak by 72 wks. Data were generated from at least three independent experiments and shown as mean ± s.d. Body weight, grip strength, and rotarod comparisons were analyzed using two-way ANOVA with Šídák post-hoc test. Significance levels are indicated as follows: *$p < 0.05$, ****$p < 0.0001$. **(D)** The Kaplan-Meier survival curve indicates comparable survival rates between cohorts. Survival was analyzed using log-rank (Mantel-Cox) test. With the exception of rotarod performance, outcomes were consistent across sexes, with no significant sex-specific differences observed.

Disease Models & Mechanisms • Supplementary information

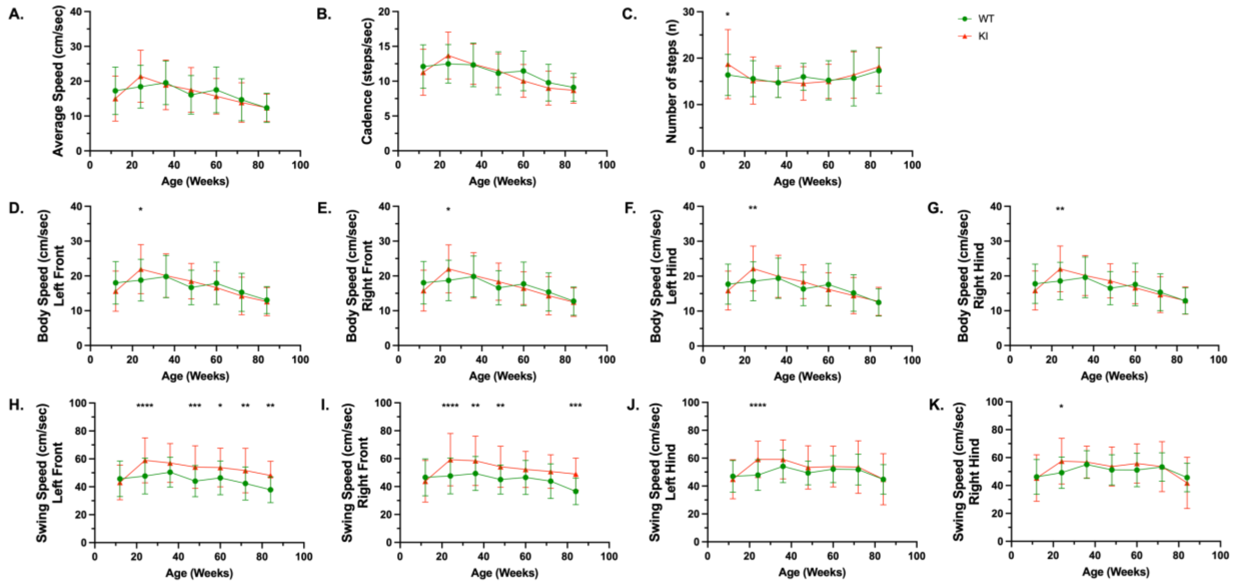

Fig. S4. Gait analysis – run characteristics and kinetic parameters. **(A)** Average speed (cm/sec) analysis. **(B)** Cadence (steps/sec) analysis. **(C)** Number of steps (n) analysis. **(D-G)** Body speed (cm/sec) analysis of forelimbs and hindlimbs. **(H-K)** Swing speed (cm/sec) analysis of forelimbs and hindlimbs. Data were generated from at least three independent experiments and shown as mean ± s.d. All comparisons were analyzed using two-way ANOVA with Šídák post-hoc test. Significance levels are indicated as follows: *$p < 0.05$, **$p < 0.01$, ***$p < 0.001$, ****$p < 0.0001$.

Disease Models & Mechanisms • Supplementary information

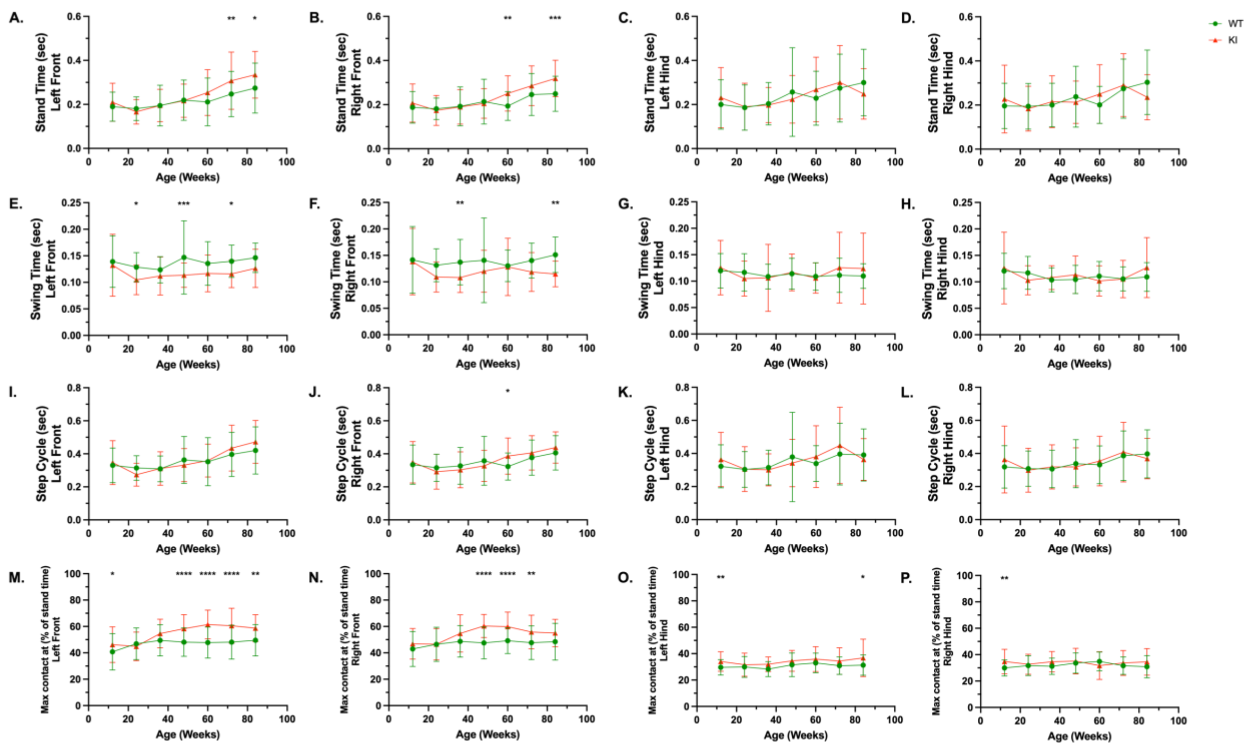

Fig. S5. Gait analysis – temporal parameters. (A-D) Stand time (sec) analysis of forelimbs and hindlimbs. **(E-H)** Swing time (sec) analysis of forelimbs and hindlimbs. **(I-L)** Step cycle (sec) analysis of forelimbs and hindlimbs. **(M-P)** Maximum contact at (% of stand time) analysis of forelimbs and hindlimbs. Data were generated from at least three independent experiments and shown as mean ± s.d. All comparisons were analyzed using two-way ANOVA with Šídák post-hoc test. Significance levels are indicated as follows: *p < 0.05, **p < 0.01, ***p < 0.001, ****p < 0.0001.

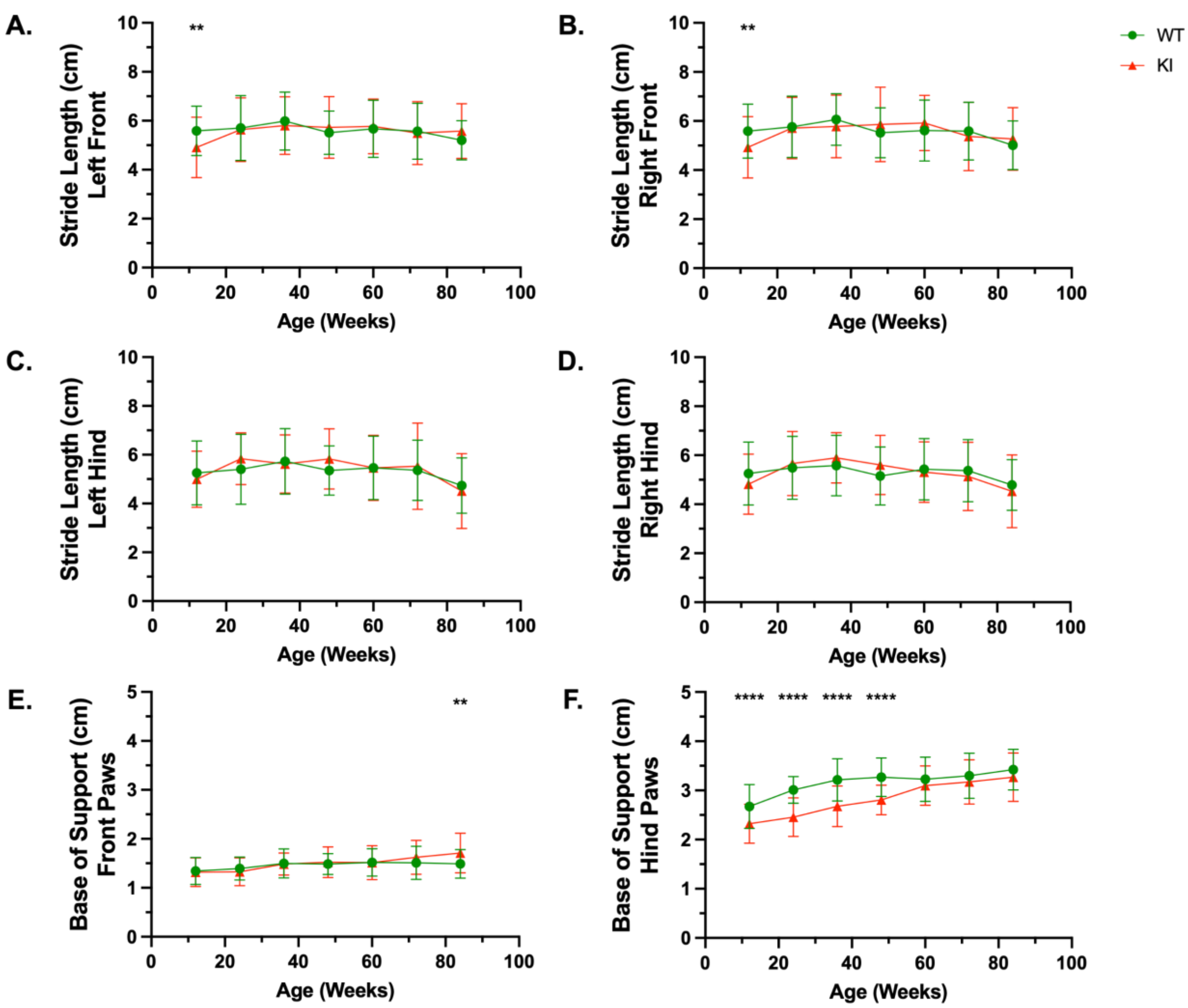

Fig. S6. Gait analysis – interlimb coordination parameters. **(A-D)** Stride length (cm) analysis in forelimbs and hindlimbs. **(E-F)** Base of support (cm) analysis of front paws and hind paws. Data were generated from at least three independent experiments and shown as mean ± s.d. All comparisons were analyzed using two-way ANOVA with Šídák post-hoc test. Significance levels are indicated as follows: **$p < 0.01$, ****$p < 0.0001$.

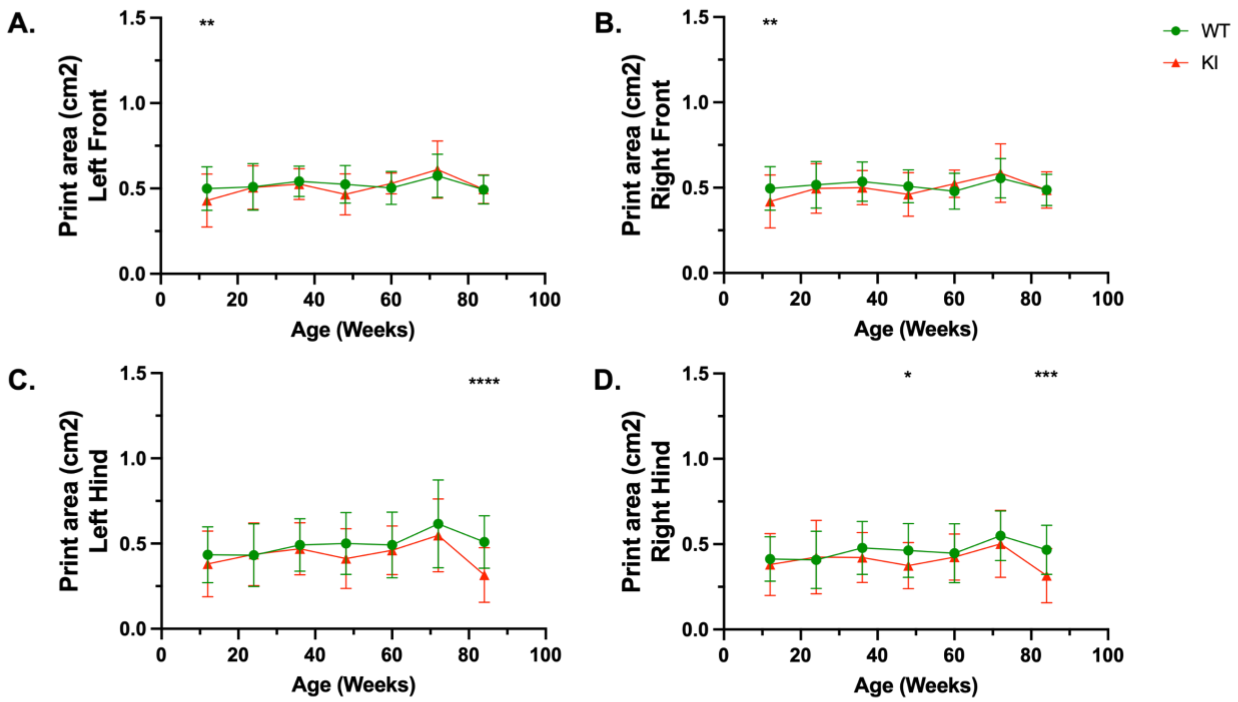

Fig. S7. Gait analysis – spatial parameters. (A-D) Analysis of print area (cm^2) in forelimbs and hindlimbs. Data were generated from at least three independent experiments and shown as mean ± s.d. All comparisons were analyzed using two-way ANOVA with Šídák post-hoc test. Significance levels are indicated as follows: *p < 0.05, **p < 0.01, ***p < 0.001, ****p < 0.0001.

Disease Models & Mechanisms • Supplementary information

A.

Murine *Gaa*$^{+/+}$ (WT) Exon 13

cactcagGGCCCTGGTCAAGACTCGGGGAACACGACCCTTTGTGAT
CTCCCGCTCAACCTTCTCGGGCCACGGCCGGTACGCTGGTCACT
GGACAGGGGATGTGCGGAGCTCTTGGGAGCATCTTGCATACTCT
GTGCCAGgtgagtacaagctgcctgcagggctctggggtcttttctgggggtctttggctgtg
ggctcaccactgcagatggtattgtgatagcttggggacatgcgccatctcatgtggcac

Murine *Gaa*$^{c.1826dupA}$ (KI) Exon 13

cactcagGGCCCTGGTCAAGACTCGGGGAACACGACCCTTTGTGAT
CTCCCGCTCAACCTTCTCGGGCCACGG**A**CGGTA**A**CGCTGGTCAC
TGGAC**T**GG**A**GATGTGCGGAGCTCTTGGGAGCATCTTGCATACTC
TGTGCCAGgtgagtacaagctgcctgcagggctctggggtcttttctgggggtctttgggctgt
gggctcaccactgcagatggtattgtgatagcttggggacatgcgccatctcatgtggcac

B.

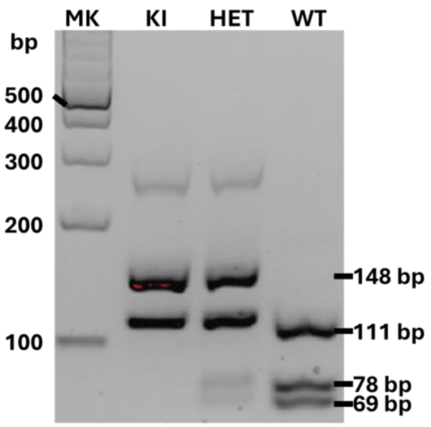

Fig. S8. Genotyping strategy for KI mice. (A) DNA sequences surrounding exon 13 of the WT murine *Gaa* gene (top) and KI murine *Gaa* gene (bottom). Lowercase letters denote intronic regions (introns 12/13), while uppercase letters indicate the coding region (exon 13). The *Rsa*I restriction enzyme recognition site is highlighted in cyan. The introduced duplication mutation (dupA) is shown in bold red, and silent mutations introduced during KI allele generation are shown in bold green, as previously described (Huang et al., 2020). **(B)** Representative gel electrophoresis of PCR amplicons following *Rsa*I digestion, resolved on a 3.5% agarose gel. WT alleles yield 111 bp, 78 bp, and 69 bp fragments. KI alleles yield 148 bp and 111 bp fragments. Heterozygous (HET) animals display all four bands: 148 bp, 111 bp, 78 bp, and 69 bp. A 100 bp DNA ladder (MK) was used as a molecular size reference.

Disease Models & Mechanisms • Supplementary information

Table S1. Impaired GAA enzyme activity in KI mice.

GAA Enzyme Activity (µg/mg protein)					
**Male**					
**Tissue**	***Gaa* Genotype**	**12wk**	**24wk**	**60wk**	**72wk**
HRT	WT	12.0±2.03	24.1±2.59	13.7±1.17	12.4±0.33
	KI	0.86±0.41****	0.88±0.13****	1.12±0.34****	0.87±0.27****
DIA	WT	9.21±0.99	8.20±0.67	9.80±0.25	9.95±3.09
	KI	0.60±0.34****	0.66±0.13****	0.68±0.09****	0.79±0.17**
GAS	WT	6.85±2.09	8.05±0.64	15.3±3.72	8.41±2.37
	KI	0.71±0.11**	0.42±0.03****	0.61±0.15**	0.52±0.11**
BRN	WT	78.7±4.40	95.2±6.92	93.7±10.8	83.9±10.4
	KI	1.03±0.05****	0.44±0.12****	0.61±0.11***	0.62±0.19***
**Female**					
**Tissue**	***Gaa* Genotype**	**12wk**	**24wk**	**60wk**	**72wk**
HRT	WT	7.90±2.53	30.9±1.45	13.6±0.52	11.9±1.53
	KI	0.61±0.16**	1.02±0.08****	0.69±0.05****	1.16±0.22***
DIA	WT	8.43±0.34	8.82±0.68	9.82±1.63	9.69±2.58
	KI	0.63±1.47****	0.68±0.07****	0.70±0.20***	0.76±0.16**
GAS	WT	3.31±1.06	7.82±0.63	9.94±0.79	6.01±0.88
	KI	0.76±0.10**	0.55±0.09****	0.48±0.07****	0.57±0.11***
BRN	WT	34.5±23.2	92.8±14.5	85.9±2.89	75.9±3.27
	KI	0.94±0.07*	0.51±0.08***	0.83±0.15****	0.44±0.05****

GAA enzyme activity levels were significantly reduced (red) in heart (HRT), diaphragm (DIA), gastrocnemius (GAS), and brain (BRN) of KI mice compared to WT controls throughout all assessed timepoints. Sample sizes (male - WT/KI): 12 wk = 4/4; 24 wk = 3/3; 60 wk = 3/3; 72 wk = 6/6. Sample sizes (female - WT/KI): 12 wk = 4/4; 24 wk = 3/3; 60 wk = 3/3; 72 wk = 3/3. Data were generated from at least three independent experiments and shown as mean ± s.d. Statistical comparisons for each tissue and corresponding time point were performed using two-tailed unpaired *t*-tests. Significance levels are indicated as follows: *p<0.05, **p<0.01, ***p<0.001, ****p<0.0001. No differences were observed when stratified by gender.

Disease Models & Mechanisms • Supplementary information

Table S2. Glycogen accumulation in cardiac and skeletal muscles of KI mice.

Glycogen Content (µmol/mg protein)					
**Male**					
**Tissue**	***Gaa* Genotype**	**12wk**	**24wk**	**60wk**	**72wk**
HRT	WT	0.00±0.00	1.69±1.51	1.07±1.85	0.38±0.67
	KI	238±22.3****	167±45.8**	90.4±15.5***	139±59.5*
DIA	WT	1.85±2.27	0.00±0.00	5.61±2.98	1.74±1.93
	KI	73.5±12.8****	83.3±5.99****	120±11.1****	117±15.5***
GAS	WT	0.98±1.13	1.31±1.56	2.82±2.35	6.02±1.25
	KI	49.2±9.97****	44.9±9.80**	47.5±15.2**	69.8±10.0***
BRN	WT	0.00±0.00	0.84±1.43	0.00±0.00	0.25±0.44
	KI	27.7±6.44***	12.2±15.7^{ns}	59.5±2.63****	52.8±8.15***
**Female**					
**Tissue**	***Gaa* Genotype**	**12wk**	**24wk**	**60wk**	**72wk**
HRT	WT	1.29±1.49	1.64±1.48	0.00±0.00	0.15±0.27
	KI	203±50.6***	201±29.0***	137±35.3**	50.0±9.75*
DIA	WT	0.00±0.00	0.53±0.92	0.85±1.47	1.38±2.39
	KI	52.7±10.7****	92.2±12.9***	127±10.2****	108±4.09****
GAS	WT	2.19±0.65	2.62±3.49	1.28±1.11	4.15±4.76
	KI	46.1±6.59****	57.1±7.44***	38.1±23.8^{ns}	85.5±31.0*
BRN	WT	0.20±0.41	0.00±0.00	0.36±0.43	0.00±0.00
	KI	22.4±3.97****	29.2±3.26***	68.4±14.4**	47.3±6.44***

Glycogen content levels were markedly increased (red) in heart (HRT), diaphragm (DIA), gastrocnemius (GAS), and brain (BRN) of KI mice compared to WT controls throughout the study duration. Sample sizes (male - WT/KI): 12 wk = 4/4; 24 wk = 3/3; 60 wk = 3/3; 72 wk = 6/6. Sample sizes (female - WT/KI): 12 wk = 4/4; 24 wk = 3/3; 60 wk = 3/3; 72 wk = 3/3. Data were generated from at least three independent experiments and shown as mean ± s.d. Statistical comparisons for each tissue and corresponding time point were performed using two-tailed unpaired *t*-tests. Significance levels are indicated as follows: *p<0.05, **p<0.01, ***p<0.001, ****p<0.0001. No differences were observed when stratified by gender.

Table S3. Gait analysis – category and parameter list

Category	Run Parameter
Run Characteristics & Kinetic Parameters	Average Speed
	Cadence
	Number of Steps
	Body Speed
	Swing Speed
Temporal Parameters	Stand Time
	Swing Time
	Step Cycle
	Max Contact at (% of Stand Time)
Interlimb Coordination Parameters	Stride Length
	Base of Support
Spatial Parameters	Print Area
